# Diclofenac Ion Hydration: Experimental and Theoretical Search for Anion Pairs

**DOI:** 10.3390/molecules27103350

**Published:** 2022-05-23

**Authors:** Anastasia V. Shishkina, Alexander A. Ksenofontov, Nikita V. Penkov, Mikhail V. Vener

**Affiliations:** 1 Department of Physics and Engineering Environmental Protection, Northern (Arctic) Federal University, 163001 Arkhangelsk, Russia; a.shishkina@narfu.ru; 2 G.A. Krestov Institute of Solution Chemistry RAS, 153045 Ivanovo, Russia; kaa@isc-ras.ru; 3 Biologically Active Terpenoids Laboratory, Kazan Federal University, 18 Kremlevskaya Street, 420008 Kazan, Russia; 4 Federal Research Center “Pushchino Scientific Center for Biological Research of the Russian Academy of Sciences”, Institute of Cell Biophysics RAS, 142290 Pushchino, Russia; nvpenkov@psn.ru; 5 Kurnakov Institute of General and Inorganic Chemistry RAS, Leninskii Prosp. 31, 119991 Moscow, Russia

**Keywords:** classical MD simulations, intermolecular hydrogen bond, DFT and TDDFT computations, Raman, infrared and UV spectroscopy, intramolecular charge transfer

## Abstract

Self-assembly of organic ions in aqueous solutions is a hot topic at the present time, and substances that are well-soluble in water are usually studied. In this work, aqueous solutions of sodium diclofenac are investigated, which, like most medicinal compounds, is poorly soluble in water. Classical MD modeling of an aqueous solution of diclofenac sodium showed equilibrium between the hydrated anion and the hydrated dimer of the diclofenac anion. The assignment and interpretation of the bands in the UV, NIR, and IR spectra are based on DFT calculations in the discrete-continuum approximation. It has been shown that the combined use of spectroscopic methods in various frequency ranges with classical MD simulations and DFT calculations provides valuable information on the association processes of medical compounds in aqueous solutions. Additionally, such a combined application of experimental and calculation methods allowed us to put forward a hypothesis about the mechanism of the effect of diclofenac sodium in high dilutions on a solution of diclofenac sodium.

## 1. Introduction

Self-assembly of organic ions in aqueous solutions is a hot topic at present [1,2,3,4,5]. Organic ions, which are readily soluble in water, are usually studied: guanidinium and arginine ions [1,2,3], protonated acridine cations [4], etc. [5,6]. The driving force of the aggregation of identically charged organic ions in aqueous solutions is solvation free energy arising from the adaptation of the hydrogen bond (H-bond) network of water to the solute [7]. Another critically important role of water is hydration of the charged groups of organic ions [1,2,3]. These water molecules are an integral part of these hydrophobic assemblies [8]. This fact significantly complicates any modelling of the structure and properties of such aggregates [9]. The good solubility of glycine and simple organic molecules in water suggests the use of relatively small cells (a solute and ~100 water molecules) [10,11], which makes it possible to perform DFT-based MD simulations and to evaluate IR spectra of such systems including the THz region [12]. Relatively large organic molecules [13,14,15] and most zwitterions of amino acids [16] require the use of much larger cells (a solute and ~>1000 water molecules), which implies performance of computer simulations with classical force fields. Although such studies sometimes calculate the IR spectra of solutes [17,18], more reliable results can be obtained using the polarizable force fields [19,20], which take into account the dipolar couplings between the solute and the solvent [10]. At present, the development of polarizable force fields is underway [21], and classical force fields have been used in this work.

The discrete-continuum model [22] is the most convenient approach to the evaluation of the vibrational frequencies of molecules and ions in aqueous solutions [23]. A serious drawback of the model is the uncertainty about the minimum number of water molecules required to simulate the first hydration shell in diluted aqueous solutions [24]. This number can be estimated based on the computer simulations with classical force fields [25,26] or some combined approaches [27]. Vibrational spectroscopy in the range of 400–4000 cm^−1^ in conjunction with the discrete-continuum model provides detailed information about the first hydration shell structure of the charged groups of organic ions [28,29,30]. However, it is not always directly related to aggregation processes in aqueous solutions. Valuable information about intermolecular interactions is provided by near-infrared (NIR) spectroscopy [31]. H-bonding effects on the wavenumbers and absorption intensities of the OH overtones of alcohol in aprotic solvents [32], dangling OH vibrations of water molecules in aqueous solutions of aprotic polar compounds [32], etc., [33] are usually investigated. The study of the first hydration shell of aqueous solutions of organic molecules with unshared electron pairs by NIR was also carried out [34,35]. The interpretation of the results obtained is hampered by the complexity of the calculations and the need to take into account anharmonic effects [31].

Low-frequency Raman spectroscopy is widely used in the study of intermolecular interactions in condensed phases. The focus is on molecular crystals [36,37,38], while relatively simple molecules are usually considered in aqueous solutions [12,39]. Difficulties with the interpretation of the obtained spectra (<200 cm^−1^) are due to two reasons. First, in organic molecules with a non-planar structure, low-frequency vibrations are a mixture of inter- and intramolecular vibrations [40]. Second, intermolecular H-bonds in this region rarely exhibit characteristic vibrations [41,42,43].

It can be concluded that there is no specific frequency range where intermolecular vibrations would appear due to the processes of association of hydrophobic ensembles. The term “hydrophobic ensemble” refers to a polyatomic organic ion and several water molecules forming H-bonds with the charged group of this ion. For each specific system, it is necessary to calculate IR and Raman spectra in the range of 10–8000 cm^−1^ and try to detect intermolecular vibrations attributed to the association (dimerization) processes.

From the experimental point of view, the main source of information about the aggregation of aromatic compounds is UV/Vis spectroscopy [4,44,45,46,47]. This process often occurs due to the so-called π-stacking interactions [48]. If charge-transfer complexes are not formed [49,50], then the shifts of the absorption/emission bands during association may not be large, since the energy of π-stacking interactions is modest [51,52]. This makes it difficult to use UV/Vis spectroscopy to distinguish between monomeric and dimeric hydrophobic assemblies.

Many medicinal compounds are poorly soluble in water and their self-assembly has been much less well-studied. We chose sodium diclofenac (NaDN), since its aqueous solutions had already been investigated using experimental and theoretical methods [26]. The poor solubility of NaDN makes it impossible to study concentrated solutions where ionic pairs usually predominate. On the other hand, association processes in saturated solutions are the first stage of nucleation, leading to the formation of a solid that precipitates from the solution.

In this work, we carried out an experimental study of aqueous solutions of NaDN at various concentrations (Section 4.1). In addition, we analyzed aqueous solutions of NaDN (0.63%) prepared from NaDN (1.25%) by mixing with water solutions: high dilution of NaDN (HD-NaDN) or high dilution of water (HD-water). Investigation of the position and shape of the first overtone of water molecules (6000–8000 cm^−1^), the position and intensity of two absorption maxima in the UV region (275 and 200 nm), and the IR spectrum in the range of 100–400 cm^−1^ led to the assumption about self-association of hydrated diclofenac anions. MD simulations of a 2 × 1 × 1 “supercell” containing 2 NaDN molecules and 2000 water molecules showed equilibrium between the hydrated anion and the hydrated dimer of the diclofenac anion. The assignment and interpretation of the bands in the UV, NIR, and IR spectra are based on DFT calculations in the discrete-continuum approximation. It has been shown that the combined use of spectroscopic methods in various frequency ranges with classical MD simulations and DFT calculations provides valuable information on the association processes of medical compounds in aqueous solutions.

## 2. Results

### 2.1. Classical MD Simulations

**Figure 1:**
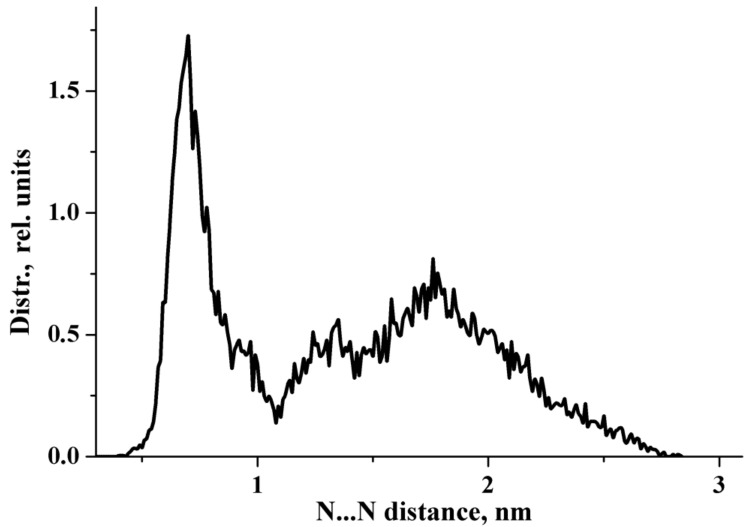
N…N distribution function obtained from the 100 ns NVT simulations.

**Figure 2:**
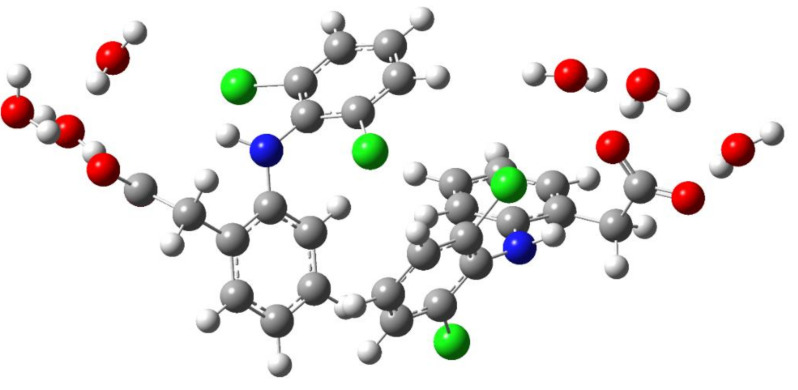
The structure of an associate of two hydrophobic ensembles (diclofenac anions with three water molecules). Red, blue, green, grey, and white circles represent oxygen, nitrogen, chlorine, carbon, and hydrogen atoms, respectively.

In the previous work [26], we focused on the interaction of the sterically hindered carboxylate group of sodium diclofenac with water. Therefore, we considered cells containing one NaDN molecule. In this study, the focus was on the solute–solute interaction, and we used a cell containing two NaDN molecules (Section 4.3). During the 100 ns NVT simulations, two DN anions spent a long time quite close to each other (Figure S1, Supplementary Materials). The N…N distribution function is shown in Figure 1. The value of ∼7 Å corresponds to an associate of two hydrophobic ensembles (dimer). A broad band in the range of ~12 to ~24 Å corresponds to solvate-separated hydrophobic ensembles (hydrated anions of diclofenac), which do not practically interact with each other. The N…N distance ∼7 Å corresponds to the structure given in Figure 2. Each diclofenac anion is shown with three water molecules that form the hydrate shell of its carboxylate (–CO_2_^−^) group. A specific characteristic of the hydration shell structure is an eight-atom ring formed by two water molecules and the –CO_2_^−^ group linked by three intermolecular H-bonds (the R_3_^3^(8) motif) [26]. The anion of diclofenac with three water molecules will be referred to as the “hydrophobic ensemble”.

Na^+^…OCO^−^ distribution function, where OCO^−^ stands for the carboxylate group of the diclofenac anion, is shown in Figure S2. It is very different from the distribution function N…N, cf. Figure 1 with Figure S2. During the MD run, the sodium cation assumes all possible configurations with respect to the carboxylate group of the diclofenac anion. The results obtained agree with the literature data [24,53]. According to Ref. [24], “in the IR spectra of dilute aqueous NaOH solutions, it is not possible to detect bands due to the interaction of the OH^−^ (nH_2_O) anions and the Na^+^ cation. This indicates the formation of ion pairs separated by the solvent”.

To evaluate the stability of this counterintuitive anion–anion pairing, the classical potential of mean force *W*(*r*) was calculated using the following formula [54]:

*W(r) = −k*_*B*_*T ln g(r)* (1)

where *k_B_* is the Boltzmann constant, *T* is the temperature, and *g*(*r*) is the radial distribution function obtained from the MD calculations. The classical potential of mean force of the N…N radial distribution function is shown in Figure S3. The generated classical potential of mean force *W*(*r*) shows the expected features of the association of polyatomic organic ions in water [1,2,3,5]. Both dimers and monomers simultaneously exist in a concentrated aqueous solution of sodium diclofenac. The results obtained are hardly applicable to the exact calculation of the equilibrium association constant, since its value significantly depends on the force field used in the simulation [55]. Due to the poor solubility of NaDN in water, it is impossible to obtain an aqueous solution where the equilibrium is strongly shifted to the left, i.e., only dimers exist. Only simulations predicted the stability of pairs of cations (anions) with respect to their forms separated by counterions and water, while experimental determination still remains a difficult task [2]. Clear evidence of strong non-covalent bonds between anions does exist in the crystalline state [56,57]. However, the nature of the binding interaction can be different in aqueous solutions and crystals.

It can be concluded that both dimers and monomers simultaneously exist in NaDN aqueous solutions.

### 2.2. DFT Computations of IR/Raman Spectra in the 10–4000 cm^−1^ Region

**Figure 3:**
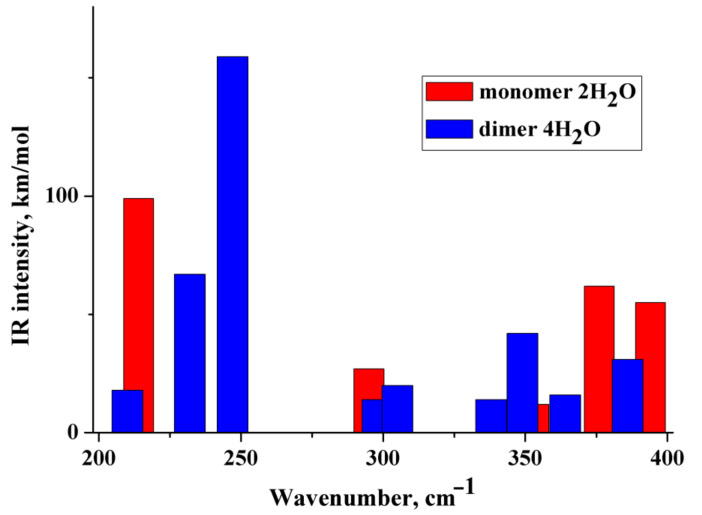
Theoretical IR spectra of the diclofenac anion hydrated with two water molecules (red columns) and dimer of the diclofenac anion hydrated with four water molecules (blue columns).

**Figure 4:**
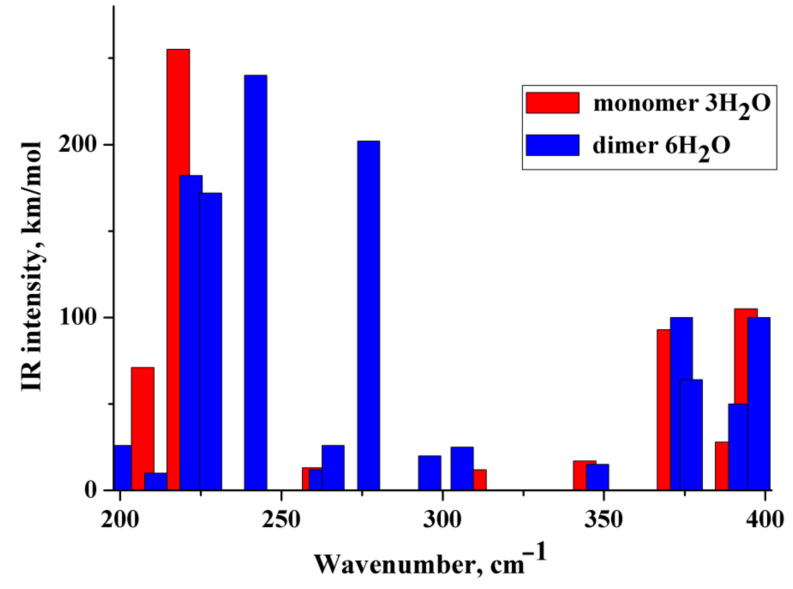
Theoretical IR spectra of the diclofenac anion hydrated with three water molecules (red columns) and dimer of the diclofenac anion hydrated with six water molecules (blue columns).

**Figure 5:**
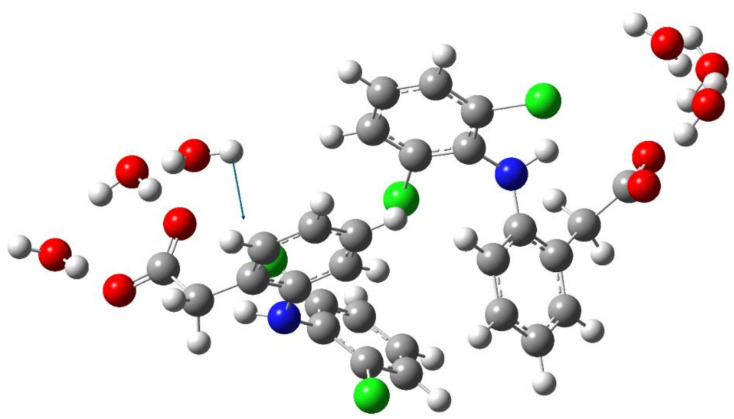
Schematic representation of the torsion vibration of the water molecule (242 cm^−1^) in dimer of the diclofenac anion hydrated with six water molecules. The arrow indicates the direction of the relative displacement of the H atom.

**Figure 6:**
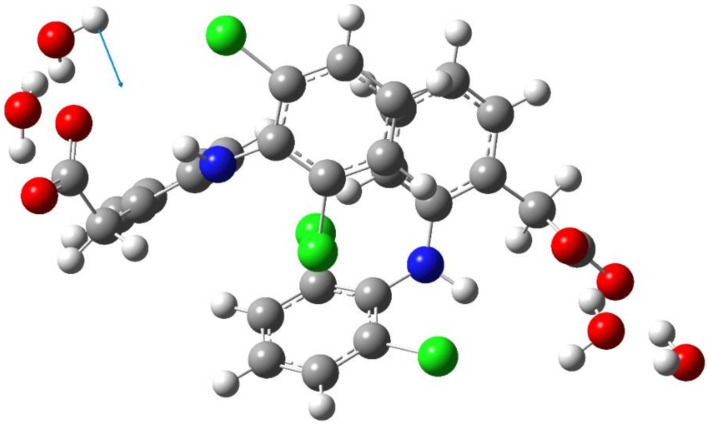
Schematic representation of the torsion vibration of the water molecule (247 cm^−1^) in dimer of the diclofenac anion hydrated with four water molecules. The arrow indicates the direction of the relative displacement of the H atom.

The spectroscopic features of the hydrated diclofenac anion (monomer) and its dimer were revealed using DFT calculations in the discrete continuum model (Section 4.4). In the case of the monomer, complexes of the diclofenac anion with two and three water molecules were taken as the initial structures [26]. The initial structure of the dimer was based on MD simulations. Each diclofenac anion molecule in the dimer is solvated by two or three water molecules. Detailed information on DFT calculations is given in Section 4.4. A careful study of the theoretical IR/Raman spectra of the hydrated monomer and dimer allows us to draw the following conclusions:

(i) The IR and Raman spectra of an anion hydrated with two and three water molecules (monomer) are practically the same. A similar situation is observed for the hydrated dimer of the diclofenac anion. (ii) Wavenumbers of IR and Raman intense vibrations of monomer and dimer practically do not differ from each other; the only exception is the region of 200–300 cm^−1^. In this region, the wavenumbers of IR-active vibrations of the dimer and monomer differ significantly (Figure 3 and Figure 4). The IR-intense band of the dimer at about 245 cm^−1^ is due to torsion vibrations of water in the hydration shell (Figure 5 and Figure 6). Its wavenumber is practically the same for the dimer hydrated by four and six water molecules.

The wavenumber of torsional vibrations of a water molecule forming the hydration shell of another anion of diclofenac dimer depends on the number of molecules that form the hydration shell. In the dimer with six water molecules, these IR intense vibrations are located between 220 and 240 cm^−1^ and around 275 cm^−1^ (Figure 4). In the monomer with three H_2_O, torsional vibrations of water molecules are located between ~200 and 220 cm^−1^. The formation of a dimer leads to a shift in the wavenumbers of torsional vibrations of water molecules to the high-frequency region. Notably, in the region of 220–300 cm^−1^, the monomer does not have intense IR bands. A similar result has been obtained for a dimer hydrated with four H_2_O (Figure 3). One intense IR vibration lies near 245 cm^−1^, and other vibrations caused by torsional vibrations of H_2_O molecules are located below 200 cm^−1^. In the case of the monomer with two H_2_O, torsion vibrations of the water molecules are below 215 cm^−1^. We conclude that the intense IR band at about 250 cm^−1^ can be considered to be a feature of the hydrated dimer of diclofenac anion. The specificity of this band lies in the temperature dependence on its IR intensity. Obviously, it should increase with decreasing temperature.

To verify the obtained results, the IR spectra of the diclofenac anion hydrated with three water molecules and the dimer of the diclofenac anion hydrated with six water molecules were calculated using wB97XD/cc-PVTZ. The polarization continuum model (PCM) was applied to simulate water medium. The dimer of diclofenac is characterized by an intense IR band at about 260 cm^−1^ due to torsional vibrations of the water molecule. Diclofenac monomer has similar vibrations below 245 cm^−1^.

### 2.3. IR Spectra in the Near-Infrared (NIR) Region: DFT Computations vs. Experiment

**Figure 7:**
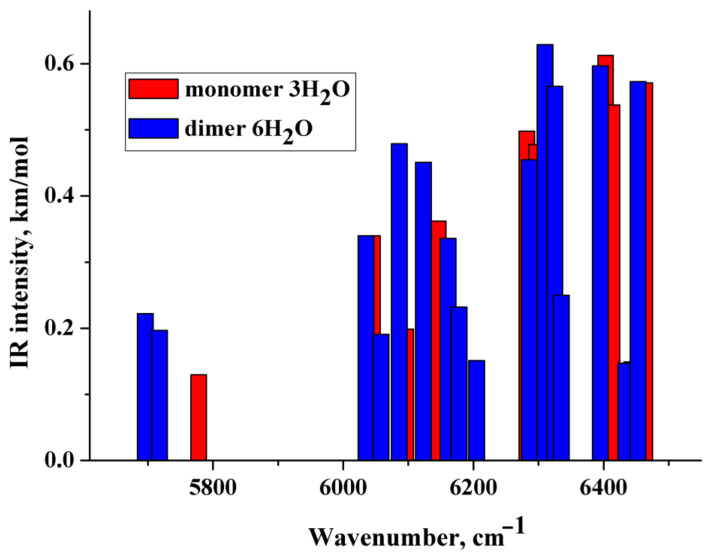
Theoretical IR spectra of the diclofenac anion hydrated with three water molecules (red columns) and dimer of the diclofenac anion hydrated with six water molecules (blue columns). Vibrations having relative IR intensities less than 0.20 are not reported.

The theoretical NIR spectra of the hydrated monomer and dimer of the diclofenac anion were performed in the anharmonic approximation (Section 4.4). The wavenumbers of the first stretching overtone O–H of the considered species are below ~6500 cm^−1^ (Figure 7 and Figure S4), i.e., lower than the wavenumbers of O−H stretches of the water molecules’ first overtone forming the first hydration shell of poly(ethylene oxide)s in an aqueous solution [34]. This is explained by the fact that the H-bonds formed by the water molecules with the CO_2_^−^ group are much shorter (stronger) than the H-bonds formed by water with the ethereal oxygen, cf. [58,59]. The lowest IR intense band is located below 5600 cm^−1^ and is associated with the first stretching overtone O–H of the water molecule of the hydrated dimers. IR intense bands caused by combination mode of the fundamental O–H stretching vibrations are located in the range of ~6000–~6500 cm^−1^ (Figure 7 and Figure S4). The obtained values of the wavenumbers are in reasonable agreement with the literature data [33]. According to Figure 7 and Figure S4, wavenumbers of IR intense vibrations of monomer and dimer practically do not differ from each other in the NIR region. Therefore, this frequency region is hardly applicable to distinguishing between monomers and dimers in NaDN aqueous solutions.

**Figure 8:**
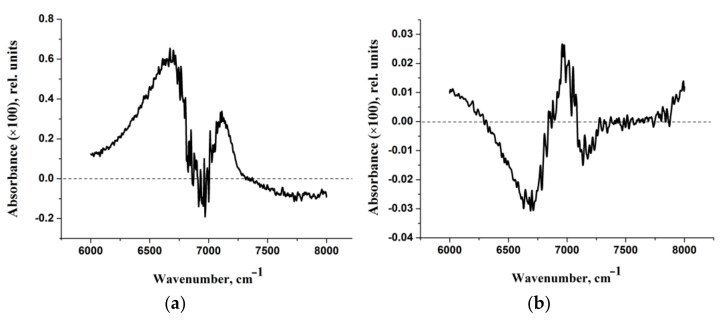
(**a**) Difference spectrum of “NaDN mixed with HD-NaDN”—“NaDN mixed with HD-water”. (**b**) Difference spectrum of “HD-NaDN”—“HD-water”.

Let us turn to the experimental data. Two types of NaDN aqueous solution (0.63%) are considered in this study. The first one was prepared from NaDN (1.25%) by mixing with HD-NaDN (Section 4.1) and will be referred to as “NaDN mixed with HD-NaDN”. The second one was prepared from NaDN (1.25%) by mixing with HD-water and will be referred to as “NaDN mixed with HD-water”. We also studied the solution of HD-NaDN and HD-water. The IR absorption spectra of the four solutions were very similar to each other (Figure S5). For a more detailed comparison, the IR absorption spectra of the four solutions were corrected (Section 4.2). There was no statistically significant difference in the position of the peak maximum in the region of 6000–8000 cm^−1^ of the adjusted spectra (Table 1). However, the areas under the peaks of the spectra “NaDN mixed with HD-NaDN” and “NaDN mixed with HD-water” differ from each other (Table 1). Next, difference spectra were obtained (Figure 8a,b). The intensity of the difference spectrum of “HD-NaDN”–“HD-water” (Figure 8b) was an order of magnitude less than the intensity of the difference spectrum of “NaDN mixed with HD-NaDN”—“NaDN mixed with HD-water” (Figure 8a). We conclude that:

(i) the results in Figure 8a,b and Figure S5 are reproducible;
(ii) there are no statistically significant differences between HD-NaDN and HD-water in terms of spectral characteristics, indicating the absence of random impurities in any of them;
(iii) IR spectra of “NaDN mixed with HD-NaDN” and “NaDN mixed with HD-water” solutions differ from each other.

Possible reasons for the differences in these spectra are discussed in Section 3.

**Table 1:** Characteristics of the adjusted spectra of four solutions (experimental groups). The result of comparison of groups (*p*-value) according to the characteristics shown in the table is indicated ^(a)^.

Spectral Characteristic	Experimental Groups			
	NaDN Mixed with HD-NaDN	NaDN Mixed with HD-Water	HD-NaDN	HD-Water
Peak position, cm^−1^ (mean ± sd)	6876 ± 4	6873 ± 6	6893 ± 2	6893 ± 2
	*p* = 0.342		*p* = 0.605	
Area (mean ± sd) ^(^^b)^	669 ± 1	664 ± 4	662 ± 1	662 ± 1
	*p* = 0.042		*p* = 0.943	

^(a)^ Statistically significant difference between the mixed experimental groups (*p* < 0.05). ^(b)^ The units are (rel. units of absorbance) cm^−1^.

Comparison of the calculated spectra (Figure 7 and Figure S4) with the experimental difference spectrum shown in Figure 8a allows us to draw the following conclusions:

- The approach used in the work makes it possible to describe only the “left” branch of the experimental difference spectrum obtained at certain dilutions of aqueous solutions of sodium diclofenac (Figure 8b). This branch of the indicated experimental spectra is “bell-shaped”, with a maximum of ~6750 cm^−1^ and a half-width of about 500 cm^−1^. The calculated spectra peak at ~6250 cm^−1^ and IR-intense bands fill the frequency range from ~6000 to ~6500 cm^−1^ (Figure 7 and Figure S4).
- It can be assumed that the “right” branch of the experimental difference spectrum is due to complex (composite) transitions caused by the simultaneous excitation of OH stretching vibrations of water molecules of the hydration shell and intramolecular vibrations, for example, asymmetric vibrations of the CO_2_^−^ group.

### 2.4. Electronic Absorption Spectra: TDDFT Computations vs. Experiment

**Figure 9:**
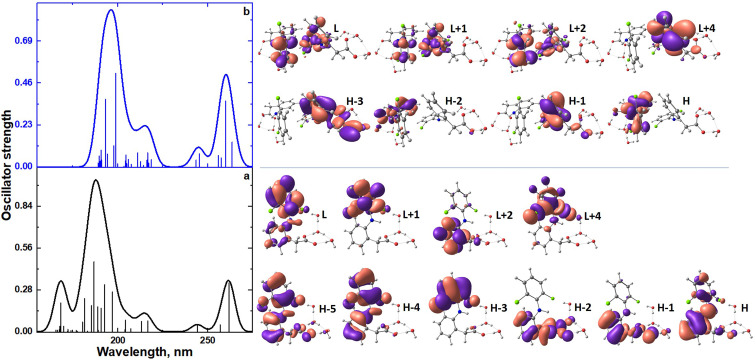
TDDFT spectra of monomer (anion diclofenac·3H_2_O) (**a**) and its dimer (**b**); the view of frontier molecular orbitals of monomer (anion diclofenac·3H_2_O) and its dimer.

The TDDFT spectra (Section 4.5) of the monomer (anion diclofenac·3H_2_O) and its dimer are compared in Figure 9 and Table 2. The monomer spectrum is characterized by the presence of nine intense bands due to H―L, H-1―L, H-2―L+2, H-2―L, H-4―L+4, H-5―L+1, H-1―L+1, H-1―L+2, H-5―L+2 electronic transitions; here, H is the highest occupied molecular orbital and L is the lowest unoccupied molecular orbital. Natural transition orbitals (NTOs) analysis for the monomer (anion diclofenac·3H_2_O) shows the main contribution to their absorption spectrum is made by H―L, H-1–L+1 electronic transitions (Tables S1 and S2). The band at 262 nm, due to the H―L electronic transition, can be caused by the partial intramolecular charge transfer from the negatively charged carboxylate group to the entire molecule (Figure 9a, Table 2). The remaining electronic transitions with higher energies are n–σ*, π–π*, and n–π* type transitions. The dimer spectrum has only five intense bands due to H―L+1, H-1―L, H-3―L, H-2―L+1, H-3―L+4 electronic transitions (Figure 9b, Table 2). In the case of a dimer, the dominant NTO pairs are H―L, H-1―L+1, H-2―L+2 (Tables S1 and S2). These bands can be attributed to partial charge transfer from one monomeric form to another. It can be assumed that the dimerization of anion diclofenac 3H_2_O contributes to an increase of the intramolecular charge transfer implementation efficiency. As a result, the oscillator strength of the band at 259–260 nm in the dimer spectrum increases compared to that of the monomer.

**Figure 10:**
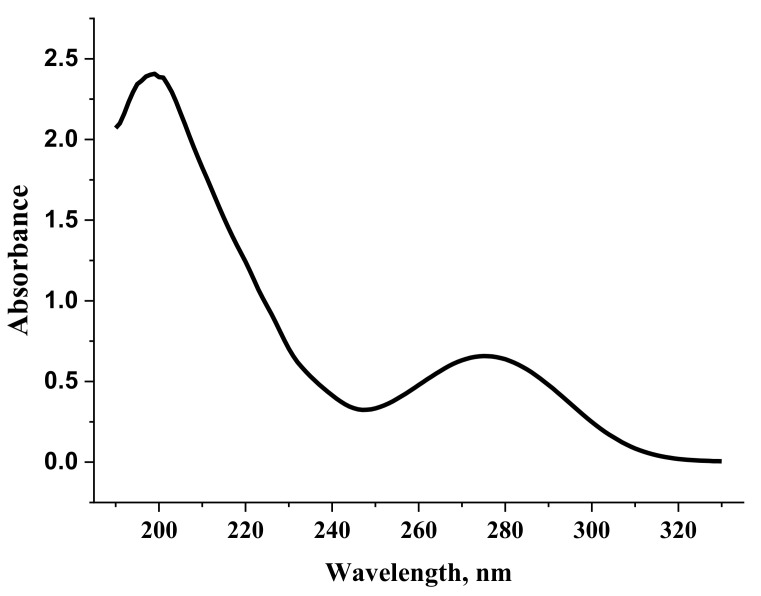
Electronic absorption spectrum of a diclofenac sodium solution (0.002%).

Compared to the monomeric form, the dimerization of anion diclofenac·3H_2_O is accompanied by a slight red shift of the absorption band maxima of the TDDFT spectrum. It should be noted that the TDDFT spectrum of the dimer lacks vertical excitation at 168 nm, which is the monomer characteristic. This band is due to the H-5—L+2 electronic transition. These spectral features can be used to identify the process of formation of dimeric forms in experimental electronic absorption spectra (Figure 10).

**Figure 11:**
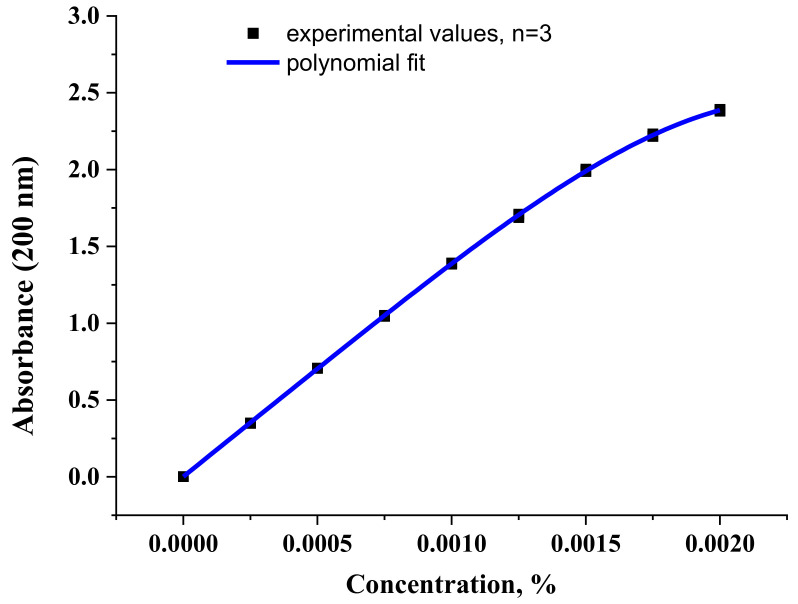
The concentration dependence of the optical density value of diclofenac sodium aqueous solution at a wavelength of 200 nm. The values obtained in the experiment and fitting with a polynomial of the 4th degree are presented.

Figure 11 shows the concentration dependence of the optical density normalized to the cuvette thickness of an aqueous solution of diclofenac sodium at a wavelength of 200 nm. In contrast to the similar dependence at 275 nm (Figure S6), a shoulder is observed at high concentrations of 200 nm. This is due to some associative processes, namely the process of dimerization of diclofenac anions. Indeed, the dimer does not absorb at a wavelength of 200 nm (Table 2) and its formation leads to a drop in the absorption intensity in the considered range. Due to the poor solubility of diclofenac sodium in water, the curve in Figure 11 is practically impossible to obtain at concentrations greater than 0.002% (Figure S7).

## 3. Discussion

Recently, the problem of infinite dilution of drug-like systems has been studied [60,61,62,63]. In this section, the physical-chemical aspects of the infinite dilution of bioactive systems will be considered, since the relationship between homeopathy and pharmacology [64] is beyond the scope of this study.

The data obtained indicate that some properties of aqueous solutions of NaDN (0.63%) depend on the method of their preparation, despite the fact that the calculated concentration of NaDN molecules in HD-NaDN and HD-water solutions approaches zero. There have already been reports in the literature about the influence of the protocol for preparing solutions on their final properties, including the ability of HD-solutions, when added to the initial solution, to change the properties of the latter [65,66,67,68,69,70]. Unfortunately, these articles only state the fact of the existence of such a phenomenon, registered by various methods, while the explanation is not given. The authors of these papers state that detailed studies are needed to explain this phenomenon. It is obvious that its explanation is possible at the atomic-molecular level and/or at the supramolecular level.

We also registered this phenomenon, showing a higher integral intensity of the IR band (about 6890 cm^−1^) in the NaDN mixed with HD-NaDN solution compared to that in the NaDN mixed with HD-water solution. At the atomic-molecular level, our finding indicates the more pronounced stretching vibrations of water molecules in NaDN mixed with HD-NaDN solution. The only factor affecting this parameter in an aqueous sample is H-bonding. It is well-known [71,72] that during the formation of an A-H…B bond, elongation (weakening) of the A-H valence bond occurs. This inevitably leads to larger amplitude of its vibrations, which is associated with an increase in the dipole moment of vibrations and an increase in the IR intensity of the absorption band due to A-H stretching vibrations [73]. In water at room temperature, almost all water molecules are linked by H-bonds; on average, there are about 3.6 H-bonds per molecule. [74]. Therefore, in this case, we can speak of a higher strength of H-bonds in the NaDN mixed with HD-NaDN sample compared to NaDN mixed with HD-water, which is consistent with the presence of a residual of a certain amount of the starting material [60,75] in the HD solution, which leads to a shift equilibrium in the “NaDN monomer–NaDN dimer” system. Dimerization also changes the properties of H-bonds (Section 2.2 and Section 2.3).

The phenomenon under consideration can also be explained at the supramolecular level—by the formation of nanoscale structures [76] or the generation of bubbles with a different size range [67,75]. The combined use of “standard” spectroscopic methods with classical MD simulations and DFT/TDDFT calculations is hardly applicable to describing these processes. This can be conducted using the methods of laser diagnostics, e.g., dynamic light scattering, laser phase microscopy, laser polarimetric scatterometry, etc. [75]. From a theoretical point of view, an adequate description of these processes requires the use of the information theory approach [77] and nucleation theory [78].

According to the results of this work, one of the physical-chemical processes occurring in aqueous solutions of diclofenac sodium is the dimerization of hydrated diclofenac anions. DFT calculations in the discrete-continuum model show that the intense IR band at about 250 cm^−1^ can be considered to be a feature of the hydrated dimer of the diclofenac anion. This band should increase with decreasing temperature. Due to the strong absorption of water in this frequency range, including the absorption of atmospheric water, the experimental detection of this band involves the use of an advanced IR spectrometer with vacuum pumping and a cooled far-IR receiver.

## 4. Materials and Methods

### 4.1. Preparing Aqueous NaDN Solutions of Various Concentrations

NaDN (Sigma-Aldrich, D6899, St. Louis, MO, USA) was used in the experiment. When preparing all test solutions, deionized water obtained using a Milli-Q^®^ Integral 5 device (Merck KGaA, Darmstadt, Germany) with resistivity of 18 MΩ × cm at 25 °C was utilized.

(1) NaDN solutions of various concentrations were prepared from NaDN (0.0125%) stock solution. The NaDN solution of each concentration was prepared in triplicate; one absorption spectrum was recorded for each replicate.
(2) Aqueous solutions of NaDN (0.63%) were prepared by mixing water solution of NaDN (1.25%) with HD of NaDN (referred to as “NaDN mixed with HD-NaDN”) or HD of water (referred to as “NaDN mixed with HD-water”). To prepare HD of NaDN, the initial NaDN solution (0.63%) was multiply serially diluted 100 times with water, with intensive shaking being performed at each step. The theoretical level of reduction in the NaDN concentration can be at least ×10^24^ times. To control the dilution technology, the water was subjected to the same procedure of serial centesimal dilutions resulting in HD of water. Each HD of NaDN, HD of water solutions, “NaDN mixed with HD-NaDN” and “NaDN mixed with HD-water” was prepared in six replicates; one IR spectrum was recorded for each replicate.

All dilutions and solutions were prepared by OOO “NPF “MATERIA MEDICA HOLDING” in sterile glass vials closed with lids (Glastechnik Gräfenroda, GmbH, Geratal, Germany). All tested solutions of NaDN were prepared by OOO “NPF “MATERIA MEDICA HOLDING” in microtubes (Eppendorf AG, Hamburg, Germany) (solutions of various concentrations) or sterile glass vials closed with lids (Glastechnik Gräfenroda, GmbH, Geratal; Germany) (mixtures with HD solutions). Mixtures with HD solutions were supplied by the manufacturer, coded, and tested blindly.

### 4.2. Spectroscopic Studies of Aqueous Solutions of Diclofenac in Various Frequency Ranges

Absorption spectra were recorded using a Shimadzu UV-1800 UV spectrophotometer (SHIMADZU CORPORATION, Kyoto, Japan) in the wavelength range from 190 to 330 nm.

The NIR spectra were measured using a Nicolet 6700 Fourier IR spectrometer (Thermo Fisher Scientific, Waltham, MA, USA) integrated with the OMNIC software (Thermo Fisher Scientific, Waltham, MA, USA). The spectra were recorded in the range of 6000–8000 cm^−1^. The OMNIC software was used to control the operation of the spectrometer, as well as to perform mathematical processing of the measured spectra. The resulting spectra were adjusted as follows: the lower point of the spectrum was set equal to zero, and the highest point was equal to one.

The coefficient of variation for the IR absorption intensity at the peak maximum is about 3%. The coefficient of variation for the area under the peak is no more than 0.6%. Statistical analysis of the data (six spectra for each type of solution) was carried out using the R language, version 3.4.0. The area under the curves in the region of 6000–8000 cm^−1^ was determined based on each absorption spectrum in the groups “HD-NaDN”, “HD-water”, “NaDN mixed with HD-NaDN”, and “NaDN mixed with HD-water”. The Shapiro–Wilk test was used for calculating the normality of the distribution of values, the Bartlett test was used for assessing the homogeneity of the variance, and the Student *t*-test or Welch *t*-test was used for comparing the groups. Each group included individual areas of the spectra of one sample in six repetitions. Thus, all possible errors in the shape of the spectra are reflected in the individual areas of the spectra and are taken into account when calculating the statistical criteria. The differences between the groups were considered statistically significant if *p* < 0.05.

### 4.3. Classical MD Simulations

In Ref. [26], a cubic cell containing 1 NaDN molecule and 1000 water molecules was prepared. It corresponds to the concentrated NaDN aqueous solution (~1.7%) [79]. The volume of the cubic cell was chosen according to the experimental density of 1.7% aqueous solutions of sodium diclofenac (1.00475 g/mL). The cube edge is 3.11667 nM. In this work, we used a 1 × 1 × 2 supercell containing 2 molecules of NaDN and 2000 water molecules. In our study, unlike Ref. [26], the main attention was paid to the solute–solute interaction. This is why atomic partial charges and van der Waals parameters of DN anion were obtained using a web-based automatic parameter generator LigParGen [80]. It provides an intuitive interface for generating OPLS-AA/1.14*CM1A-LBCC force field parameters for organic ligands, in the formats of commonly used molecular dynamics programs. The server currently accepts three different standard input formats for molecular structures: SMILES codes, PDB, and MOL files. In our case, we used PDB format that was pre-generated from cif files. The topological file of the DN-anion is given in SI.

The GROMACS code [81,82,83,84] was used to perform the MD simulations. The force field OPLS-AA [85] was used together with the SPC/E water model [86]. The simulations were carried out in the NVT (constant number, constant volume, and constant temperature) ensemble. The temperature was maintained at 298 K, employing the velocity-rescaling temperature coupling [87] with the time constant of 0.5 ps. The equations of motion were integrated using the leap-frog algorithm [88] with a time step of 0.5 fs. Long-range electrostatic interactions were calculated using the particle mesh Ewald method [89,90] (the cutoff was set at 15 Å); van der Waals and short-range interactions were truncated at 14 Å. The fluctuations of kinetic, potential, and total energy around some mean values serve as criterion for the equilibration of the systems (100 ns, a time step is 0.5 fs). For information purposes, 100 ns (a time step is 0.5 fs) simulations were performed.

### 4.4. DFT Computations

The structure, IR, and Raman spectra of the hydrated diclofenac monomer and its dimer were computed at the B3LYP-D3/6-311++G** and wB97XD/cc-PVTZ levels using Gaussian 16 software package (Gaussian, Inc., Wallingford, CT, USA) [91]. The London dispersion interactions were taken into account by introducing the D3 correction developed by Grimme et al. [92]. The scaled factors [93] were not used. The PCM model was applied to simulate water medium [94].

The near-infrared spectrum in the region of the first stretching overtone of water O−H groups was evaluated using the freq = (Anharmonic,SelectAnharmonicModes) option [91]. For differentiation in anharmonic analysis, all O-H stretch modes of water molecules of the considered hydrate complex were used.

### 4.5. TDDFT Computations

The vertical electronic transitions were computed by the TDDFT method using Gaussian 16 software package [91], employing CAMB3LYP/def2-SVP [95,96]. TDDFT analysis was performed for the first 30 singlet excited states. The results of TDDFT analysis were supplemented with natural transition orbital analysis (NTO). NTO represents the most compact orbital representation for a given single-electronic excitation. The NTO analysis was carried out for all found excited states of monomer and dimer. The PCM model was applied to simulate water medium. Visualization of the results was performed with Chemcraft 1.8 [97].

## 5. Conclusions

According to classical MD simulations, both dimers and monomers simultaneously exist in sodium diclofenac aqueous solutions. Due to the poor solubility of sodium diclofenac in water, it is impossible to obtain an aqueous solution where the equilibrium is strongly shifted to the left, i.e., only dimers exist. This complicates the experimental identification of dimers.

The following approach is proposed for studying isomerization processes in aqueous solutions of medicinal compounds: the structure of the hydration shell and the spatial orientation of molecules/ions in dimers are obtained from MD simulations. The structures of monomeric and dimeric hydrophobic ensembles are used to calculate vibrational spectra in the frequency ranges 10–4000 and 6000–8000, as well as electronic absorption/emission spectra in the UV/visible range. The resulting spectroscopic features of the dimers are verified experimentally.

The combined use of spectroscopic methods in the UV, NIR, and IR frequency ranges with classical MD simulations and DFT/TDDFT calculations provides valuable information on the association processes of medical compounds in aqueous solutions. Additionally, we suggest that the addition of HD-NaDN solution to NaDN solution causes changes in the properties of the latter at the atomic-molecular or supramolecular levels. Indirect information about dimerization is provided by studies of electronic absorption spectra in the region of ~200 nm and IR spectra in the range of 200–400 cm^−1^. We do hope that this work will promote the use of combined computational-experimental spectroscopic search for studying the association processes of medical compounds in aqueous solutions.

## Figures and Tables

**Table 2:** Results of TDDFT calculations of the monomer (anion diclofenac·3H_2_O) and its dimer.

Compound	State	Transition Nature (Contribution, %)	Wavelength, nm (eV)	*f* ^(^^*)^
Monomer	1	H―L	262 (4.73)	0.33
	9	H-1―L	196 (6.29)	0.27
	10	H-2―L+2	193 (6.43)	0.32
	11	H-2―L	191 (6.50)	0.16
	13	H-4―L+4	189 (6.57)	0.17
	14	H-5―L+1	187 (6.64)	0.47
	15	H-1―L+1	185 (6.69)	0.17
	17	H-1―L+2	181 (6.83)	0.22
	25	H-5―L+2	168 (7.37)	0.19
Dimer	1	H―L+1	264 (4.70)	0.14
	2	H-1―L	260 (4.77)	0.36
	21	H-3―L	199 (6.24)	0.51
	22	H-2―L+1	198 (6.27)	0.12
	24	H-3―L+4	193 (6.42)	0.37

* Electronic transitions with an oscillator strength *f* of more than 0.1 are given.

## Data Availability

The IR spectra and I/O files are available from the respective author upon reasonable request.

## Supplementary Materials

The following are available online at https://www.mdpi.com/article/10.3390/molecules27103350/s1, Figure S1: The distance between the nitrogen atoms of DN anions during the 100 ns NVT simulations, Figure S2: Na^+^…OCO^−^ distribution function obtained from the 100 ns NVT simulations, here OCO^−^ stands for the carboxylate group of the diclofenac anion, Figure S3: The classical potential of mean force *W*(*r*) of the N…N radial distribution function. The reaction coordinate corresponds to the distance separating the nitrogen atoms *r*(N…N) of the DN anions, Figure S4: Theoretical IR spectra of the diclofenac anion hydrated with two water molecules (red sticks) and dimer of the diclofenac anion hydrated by four water molecules (blue sticks). Vibrations having relative IR intensities less than 0.20 are not reported, Figure S5: IR spectrum of NaDN mixed with HD-NaDN, Table S1: NTOs analysis for monomer (anion diclofenac·3H_2_O) and its dimer, Table S2: Visualization of hole and particle NTOs of monomer (anion diclofenac·3H_2_O) and dimer, Figure S6: The concentration dependence of the optical density normalized to the cuvette thickness of an aqueous solution of diclofenac sodium at a wavelength 275 nm. The values obtained in the experiment and linear fit are presented, Figure S7: The concentration dependence of the optical density normalized to the cuvette thickness of an aqueous solution of diclofenac sodium at a wavelength 200 nm. Optical densities obtained at concentrations greater than 0.002% must be interpreted with care, Section S1: Topological file of the DN-anion, Section S2: Structure of the diclofenac anion hydrated with two water molecules, Section S3: Structure of the diclofenac anion hydrated with three water molecules, Section S4: Structure of dimer of the diclofenac anion hydrated with four water molecules, Section S5: Structure of dimer of the diclofenac anion hydrated with six water molecules.

## Sample Availability

Samples of the compounds are not available from the authors.
