# Supplementary material for: Diclofenac Ion Hydration: Experimental and Theoretical Search for Anion Pairs
Source: Molecules. 2022 May 23;27(10):3350. doi: 10.3390/molecules27103350 (PMC9146526; doi:10.3390/molecules27103350)
Supplement: Supplementary file 1 [file molecules-27-03350-s001.zip › molecules-1695094-supplementary.pdf]

molecules

Supplementary Materials

Diclofenac Ion Hydration: Experimental and Theoretical Search for Anion Pairs

Anastasia V. Shishkina [1], Alexander A. Ksenofontov [2,3], Nikita V. Penkov [4] and Mikhail V. Vener [5,*]

[1] Department of Physics and Engineering Environmental Protection, Northern (Arctic) Federal University, 163001 Arkhangelsk, Russia; a.shishkina@narfu.ru
[2] G.A. Krestov Institute of Solution Chemistry RAS, 153045 Ivanovo, Russia; kaa@isc-ras.ru
[3] Biologically Active Terpenoids Laboratory, Kazan Federal University, 18 Kremlevskaya Street, 420008 Kazan, Russia
[4] Federal Research Center "Pushchino Scientific Center for Biological Research of the Russian Academy of Sciences", Institute of Cell Biophysics RAS, 142290 Pushchino, Russia; nvpenkov@psn.ru
[5] Kurnakov Institute of General and Inorganic Chemistry RAS, Leninskii Prosp. 31, 119991 Moscow, Russia
* Correspondence: vener@igic.ras.ru

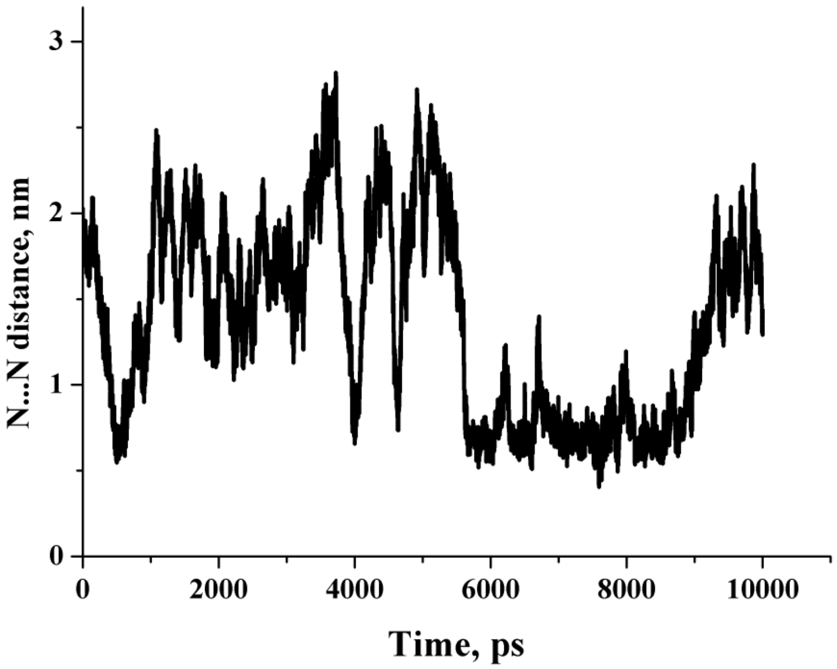

Figure S1. The distance between the nitrogen atoms of DN anions during the 100 ns *NVT* simulations.

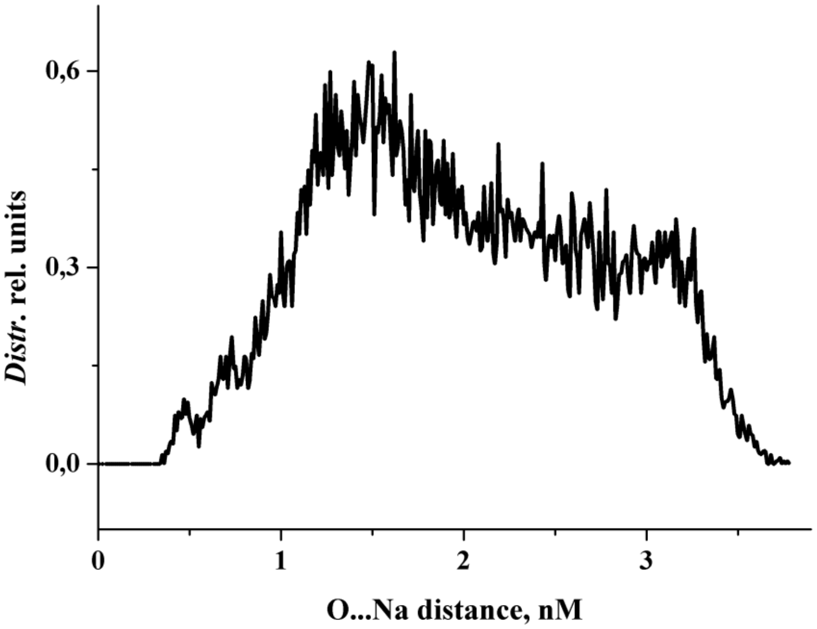

Figure S2. Na^{+}…OCO^{-} distribution function $g(r)$ obtained from the 100 ns NVT simulations, where OCO^{-} stands for the carboxylate group of the diclofenac anion.

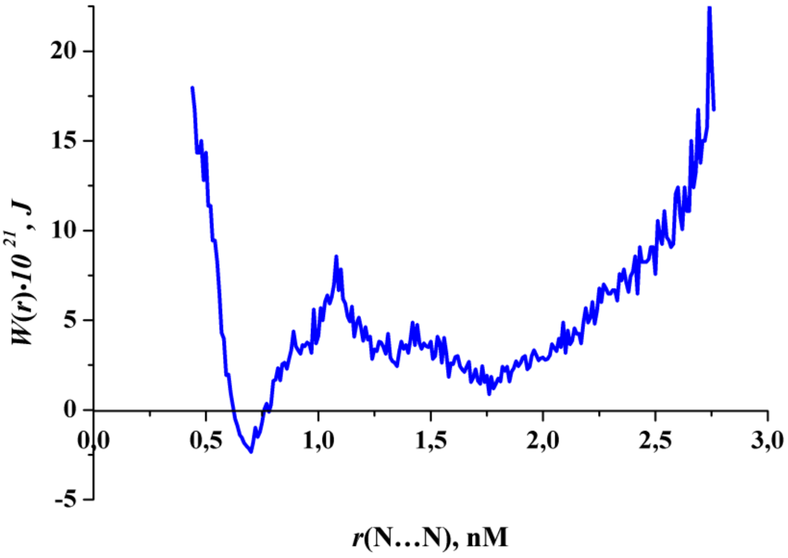

Figure S3. The classical potential of mean force $W(r)$ of the N…N distribution function. The reaction coordinate corresponds to the distance separating the nitrogen atoms r(N…N) of the DN anions.

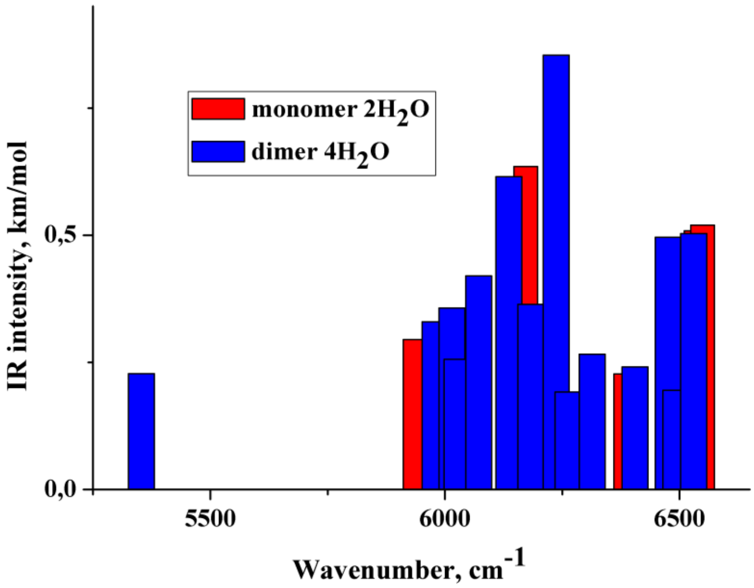

Figure S4. Theoretical NIR spectra of the diclofenac anion hydrated with two water molecules (red sticks) and dimer of the diclofenac anion hydrated by four water molecules (blue sticks). Vibrations having relative IR intensities less than 0.20 are not reported.

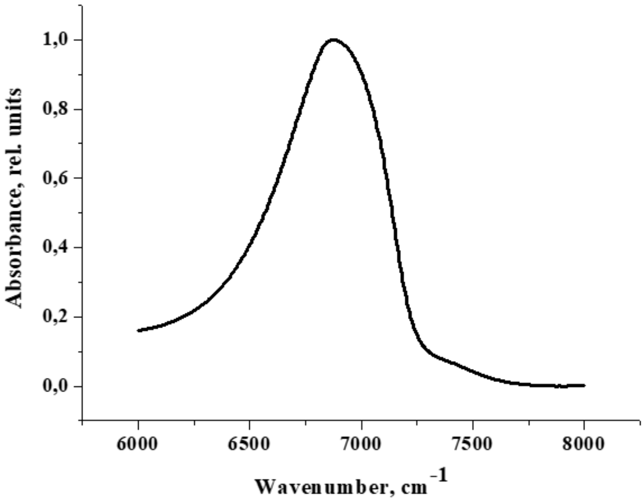

Figure S5. NIR spectrum of NaDN mixed with HD-NaDN.

Table S1. NTOs analysis for monomer (anion diclofenac·3H_2O) and its dimer.

State	Transition Nature	NTO Eigenvalue
	monomer (anion diclofenac·3H_2O)	
1	H—L	0.97
9	H—L	0.73
	H-1—L+1	0.22
10	H—L	0.64
	H-1—L+1	0.19
	H-2—L+2	0.11
11	H—L	0.66
	H-1—L+1	0.15
	H-2—L+2	0.10
13	H—L	0.41
	H-1—L+1	0.26
	H-2—L+2	0.18
14	H—L	0.67
	H-1—L+1	0.29
15	H—L	0.61
	H-1—L+1	0.27
17	H—L	0.57
	H-1—L+1	0.26
25	H—L	0.66
	H-1—L+1	0.27
	dimer	
1	H—L	0.88
2	H—L	0.87
21	H—L	0.40
	H-1—L+1	0.33
	H-2—L+2	0.13
22	H—L	0.38
	H-1—L+1	0.31
	H-2—L+2	0.13
24	H—L	0.42
	H-1—L+1	0.25

Table S2. Visualization of hole and particle NTOs of monomer (anion diclofenac·3H_2O) and its dimer.

State	Molecular Orbitals	
1	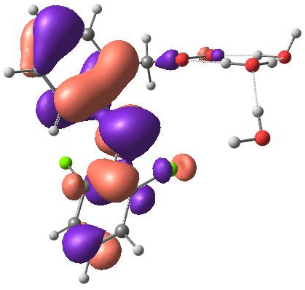 H	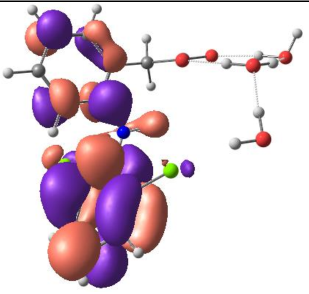 L
9	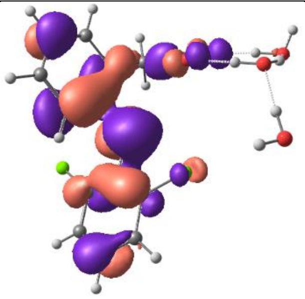 H-1	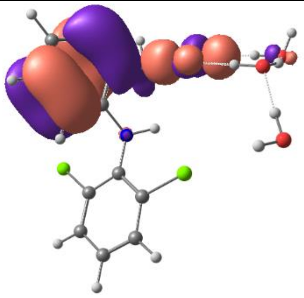 H
	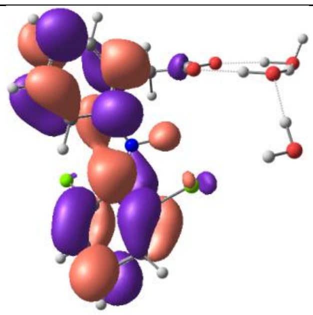 L	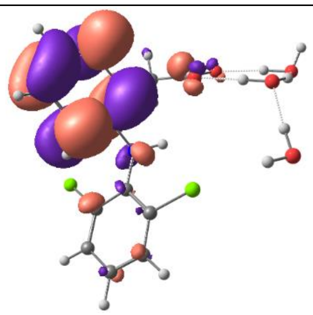 L+1
10	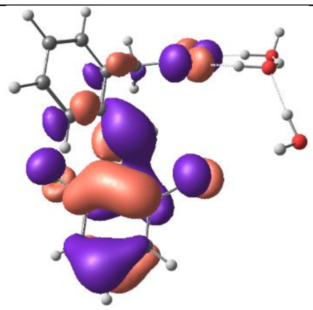 H-2	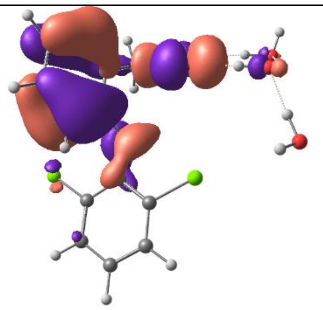 H-1
	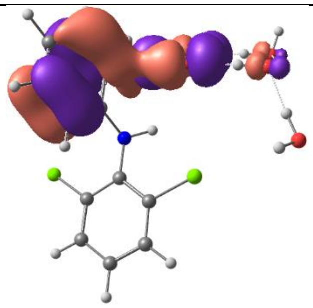 H	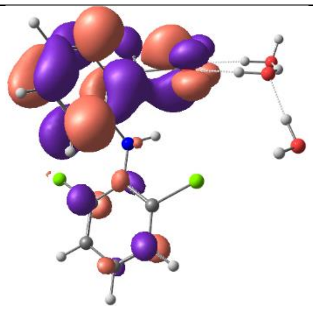 L

	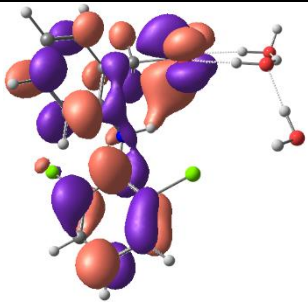<br>L+1	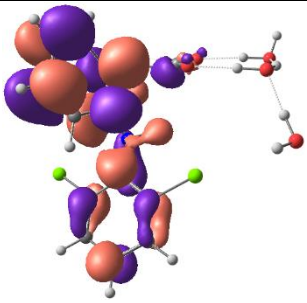<br>L+2
11	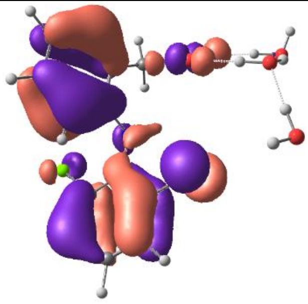<br>H-2	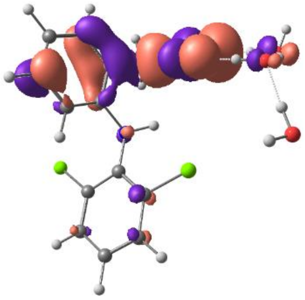<br>H-1
	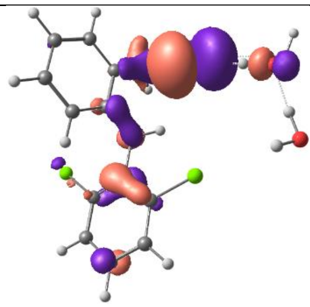<br>H	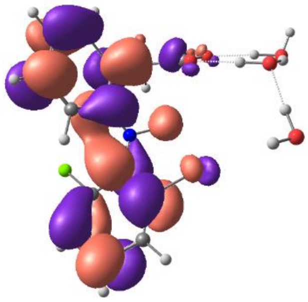<br>L
	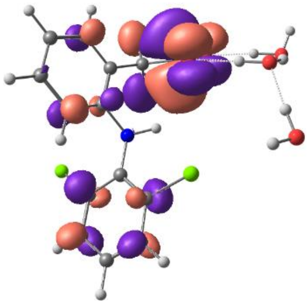<br>L+1	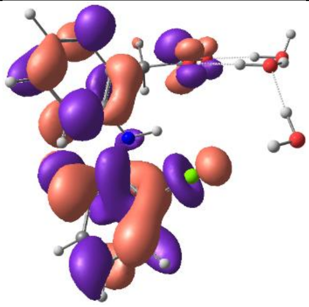<br>L+2
13	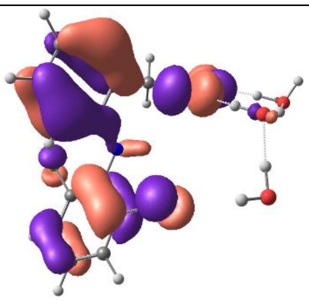<br>H-2	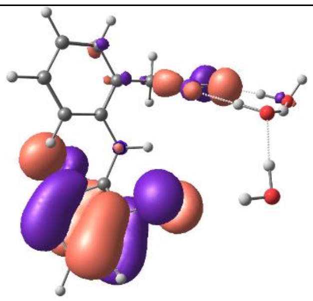<br>H-1

	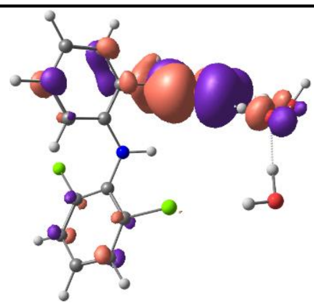<br>H	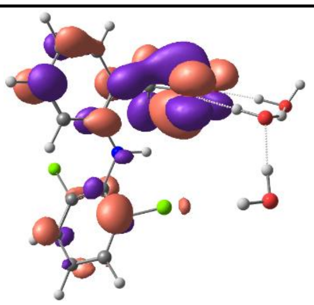<br>L
	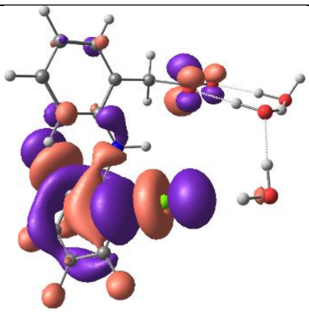<br>L+1	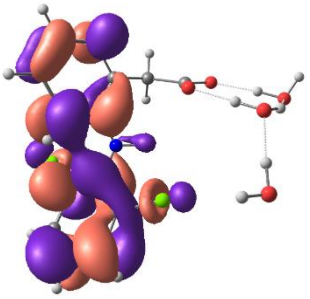<br>L+2
14	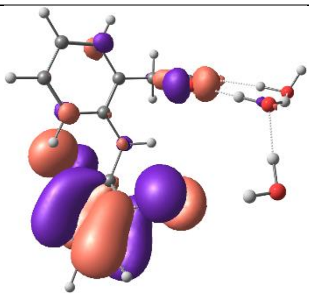<br>H-1	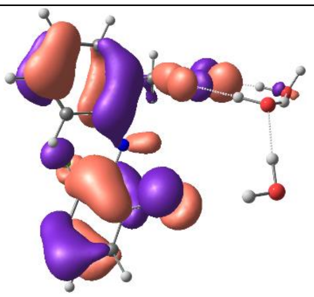<br>H
	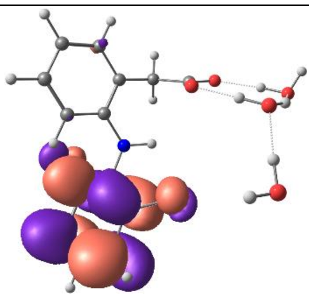<br>L	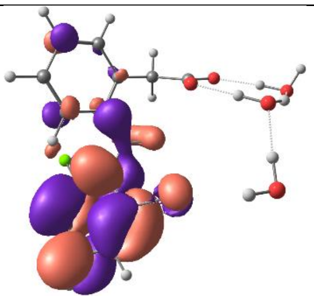<br>L+1
15	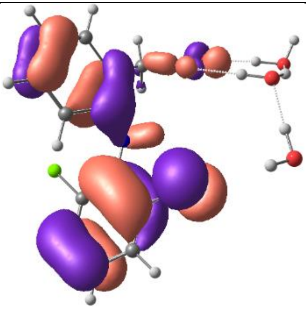<br>H-1	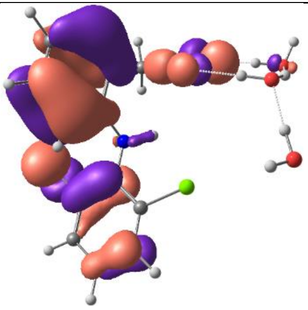<br>H

	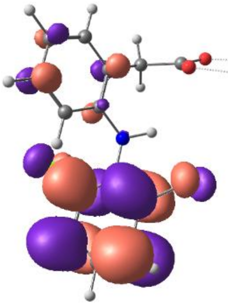 L	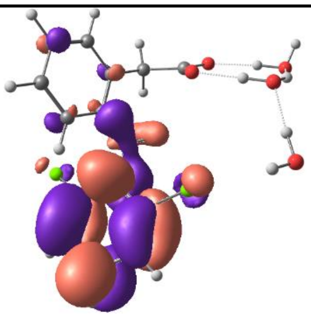 L+1
17	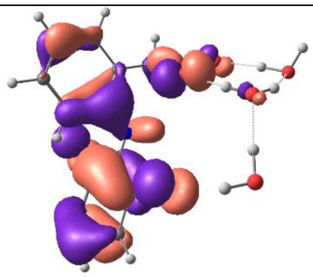 H-1	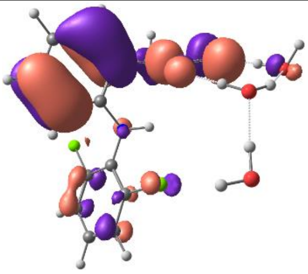 H
	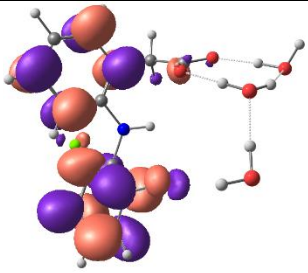 L	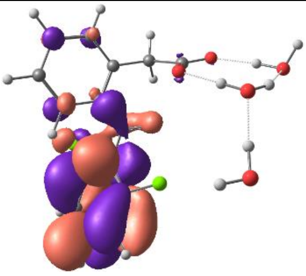 L+1
25	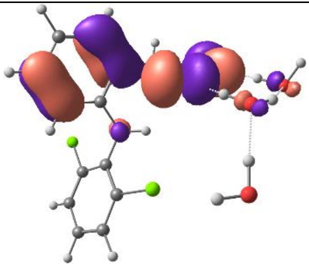 H-1	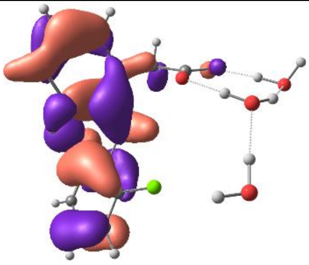 H
	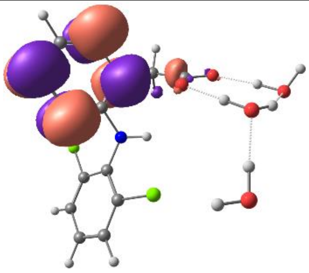 L	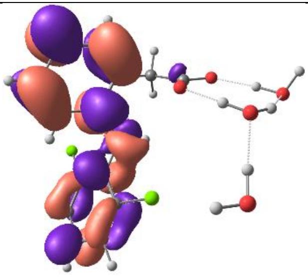 L+1

1	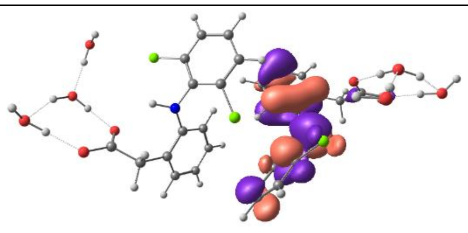 H	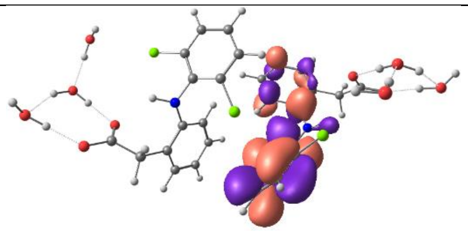 L

2	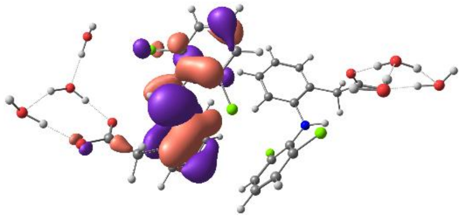<br>H	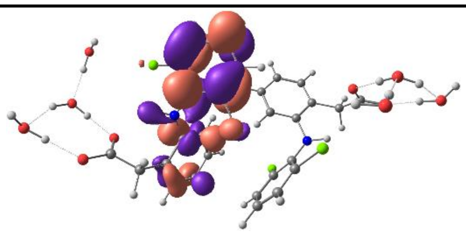<br>L
21	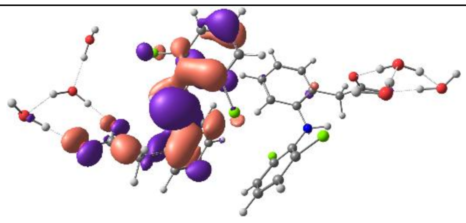<br>H-2	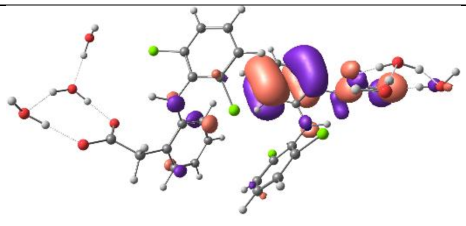<br>H-1
	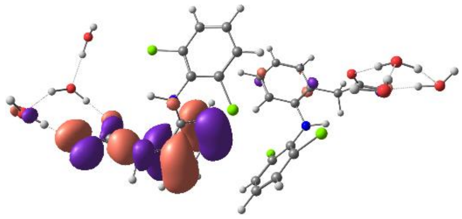<br>H	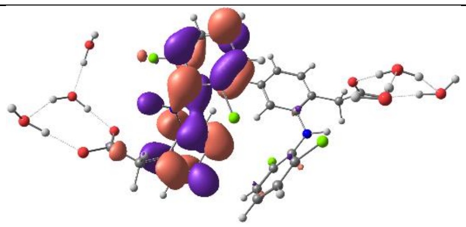<br>L
	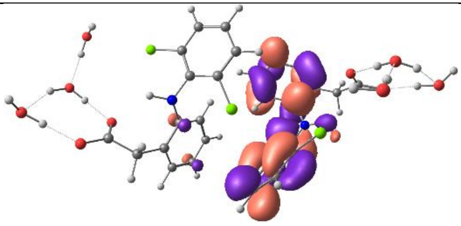<br>L+1	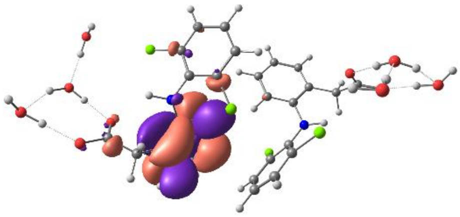<br>L+2
22	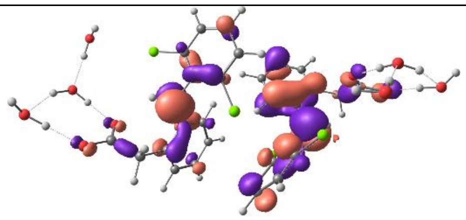<br>H-2	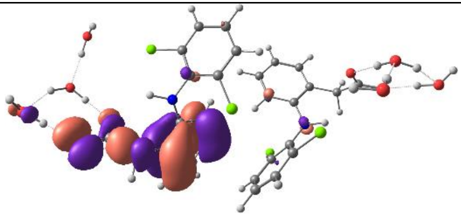<br>H-1
	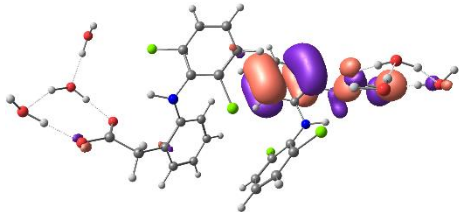<br>H	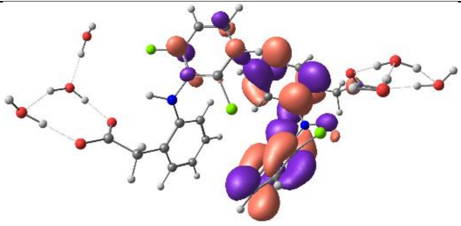<br>L
	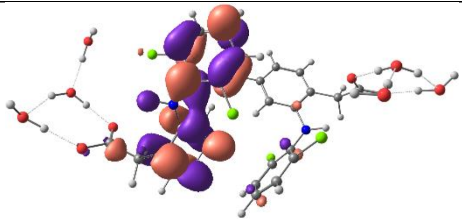<br>L+1	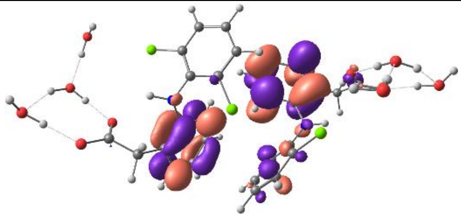<br>L+2

24	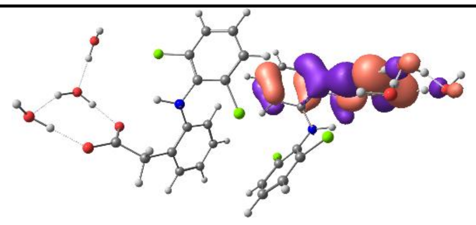 H-1	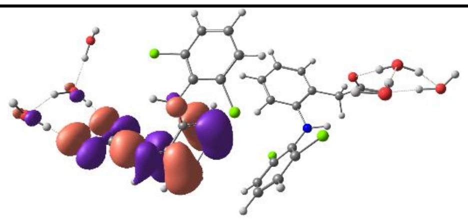 H
	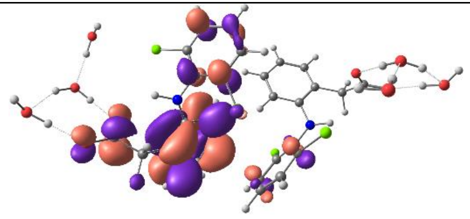 L	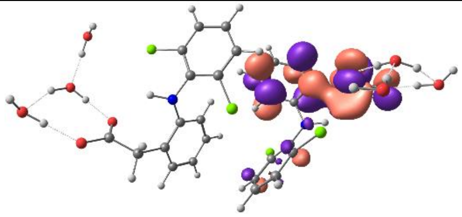 L+1

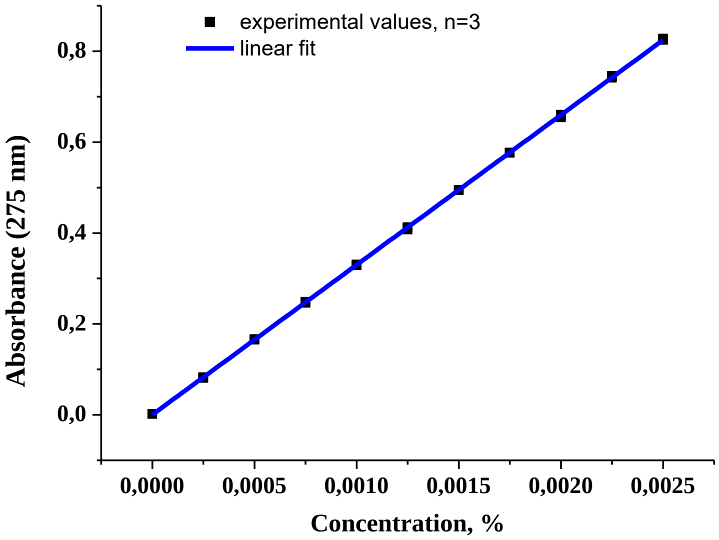

Figure S6. The concentration dependence of the optical density normalized to the cuvette thickness of an aqueous solution of diclofenac sodium at a wavelength 275 nm. The values obtained in the experiment and linear fit are presented.

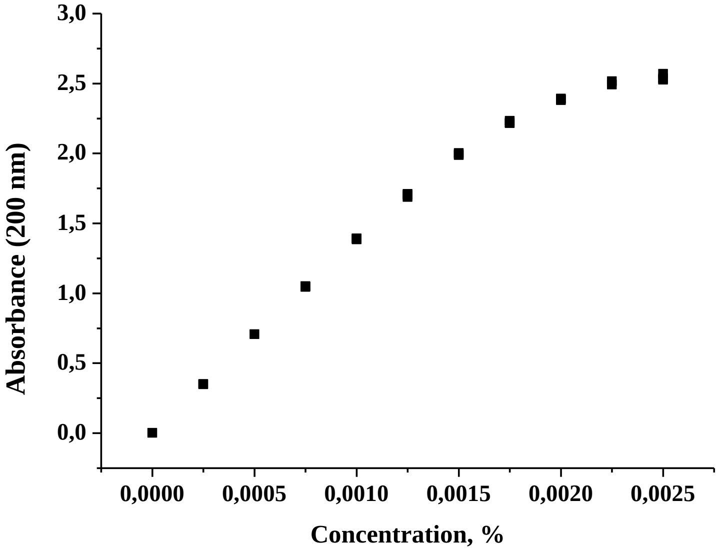

Figure S7. The concentration dependence of the optical density normalized to the cuvette thickness of an aqueous solution of diclofenac sodium at a wavelength 200 nm. Optical densities obtained at concentrations greater than 0.002% must be interpreted with care.

Section S1. Topological file of the DN-anion.

```
; GENERATED BY LigParGen Server
; Jorgensen Lab @ Yale University
;
[ moleculetype ]
; Name               nrexcl
UNK                  3


[ atoms ]
;   nr       type  resnr residue  atom   cgnr     charge       mass
     1   opls_800      1    UNK    O00      1    -0.6139    15.9990
     2   opls_801      1    UNK    C01      1     0.4329    12.0110
     3   opls_802      1    UNK    O02      1    -0.6139    15.9990
     4   opls_803      1    UNK    C03      1    -0.2153    12.0110
     5   opls_804      1    UNK    C04      1    -0.0287    12.0110
```

6	opls_805	1	UNK	C05	1	-0.1288	12.0110
7	opls_806	1	UNK	C06	1	-0.1343	12.0110
8	opls_807	1	UNK	C07	1	-0.1635	12.0110
9	opls_808	1	UNK	C08	1	-0.1102	12.0110
10	opls_809	1	UNK	C09	1	0.1217	12.0110
11	opls_810	1	UNK	N0A	2	-0.6145	14.0070
12	opls_811	1	UNK	C0B	3	0.2398	12.0110
13	opls_812	1	UNK	C0C	3	-0.0574	12.0110
14	opls_813	1	UNK	Cl0	3	-0.0438	35.4500
15	opls_814	1	UNK	C0E	3	-0.0994	12.0110
16	opls_815	1	UNK	C0F	3	-0.1771	12.0110
17	opls_816	1	UNK	C0G	3	-0.1034	12.0110
18	opls_817	1	UNK	C0H	3	-0.0712	12.0110
19	opls_818	1	UNK	Cl1	3	-0.0605	35.4500
20	opls_819	1	UNK	H0J	1	0.0780	1.0080
21	opls_820	1	UNK	H0K	1	0.0780	1.0080
22	opls_821	1	UNK	H0M	1	0.1361	1.0080
23	opls_822	1	UNK	H0N	1	0.1113	1.0080
24	opls_823	1	UNK	H0O	1	0.1116	1.0080
25	opls_824	1	UNK	H0P	1	0.1238	1.0080
26	opls_825	1	UNK	H0Q	2	0.4126	1.0080
27	opls_826	1	UNK	H0R	3	0.1353	1.0080
28	opls_827	1	UNK	H0S	3	0.1248	1.0080
29	opls_828	1	UNK	H0T	3	0.1300	1.0080

[bonds]

2	1	1	0.1250 548940.800
3	2	1	0.1250 548940.800
4	2	1	0.1522 265265.600
5	4	1	0.1510 265265.600

6	5	1	0.1400 392459.200
7	6	1	0.1400 392459.200
8	7	1	0.1400 392459.200
9	8	1	0.1400 392459.200
10	5	1	0.1400 392459.200
11	10	1	0.1340 402500.800
12	11	1	0.1340 402500.800
13	12	1	0.1400 392459.200
14	13	1	0.1725 251040.000
15	13	1	0.1400 392459.200
16	15	1	0.1400 392459.200
17	16	1	0.1400 392459.200
18	12	1	0.1400 392459.200
19	18	1	0.1725 251040.000
20	4	1	0.1090 284512.000
21	4	1	0.1090 284512.000
22	6	1	0.1080 307105.600
23	7	1	0.1080 307105.600
24	8	1	0.1080 307105.600
25	9	1	0.1080 307105.600
26	11	1	0.1010 363171.200
27	15	1	0.1080 307105.600
28	16	1	0.1080 307105.600
29	17	1	0.1080 307105.600
10	9	1	0.1400 392459.200
18	17	1	0.1400 392459.200

[angles]

; ai aj ak funct c0 c1 c2 c3

1	2	3	1	126.000	669.440
1	2	4	1	117.000	585.760
2	4	5	1	112.000	527.184
4	5	6	1	120.000	585.760
5	6	7	1	120.000	527.184
6	7	8	1	120.000	527.184
7	8	9	1	120.000	527.184
4	5	10	1	120.000	585.760
5	10	11	1	120.000	585.760
10	11	12	1	116.000	418.400
11	12	13	1	120.000	585.760
12	13	14	1	120.000	627.600
12	13	15	1	120.000	527.184
13	15	16	1	120.000	527.184
15	16	17	1	120.000	527.184
11	12	18	1	120.000	585.760
12	18	19	1	120.000	627.600
2	4	20	1	109.500	292.880
2	4	21	1	109.500	292.880
5	6	22	1	120.000	292.880
6	7	23	1	120.000	292.880
7	8	24	1	120.000	292.880
8	9	25	1	120.000	292.880
10	11	26	1	116.000	292.880
13	15	27	1	120.000	292.880
15	16	28	1	120.000	292.880
16	17	29	1	120.000	292.880
18	17	29	1	120.000	292.880
16	17	18	1	120.000	527.184

7	6	22	1	120.000	292.880
6	5	10	1	120.000	527.184
12	18	17	1	120.000	527.184
5	10	9	1	120.000	527.184
17	16	28	1	120.000	292.880
14	13	15	1	120.000	627.600
12	11	26	1	116.000	292.880
16	15	27	1	120.000	292.880
8	7	23	1	120.000	292.880
3	2	4	1	117.000	585.760
10	9	25	1	120.000	292.880
17	18	19	1	120.000	627.600
5	4	21	1	109.500	292.880
5	4	20	1	109.500	292.880
9	10	11	1	120.000	585.760
8	9	10	1	120.000	527.184
20	4	21	1	107.800	276.144
13	12	18	1	120.000	527.184
9	8	24	1	120.000	292.880

[dihedrals]

; IMPROPER DIHEDRAL ANGLES

; ai aj ak al funct c0 c1 c2 c3 c4 c5

15	13	12	14	4	180.000	10.460	2
10	5	4	6	4	180.000	10.460	2
18	12	11	13	4	180.000	10.460	2
4	2	1	3	4	180.000	43.932	2
19	18	12	17	4	180.000	10.460	2

22	6	5	7	4	180.000	10.460	2
24	8	7	9	4	180.000	10.460	2
23	7	6	8	4	180.000	10.460	2
27	15	13	16	4	180.000	10.460	2
28	16	15	17	4	180.000	10.460	2
29	17	16	18	4	180.000	10.460	2
25	9	8	10	4	180.000	10.460	2
11	10	5	9	4	180.000	10.460	2

[dihedrals]

; PROPER DIHEDRAL ANGLES

; ai	aj	ak	al	funct	c0	c1	c2	c3	c4	c5
8	9	10	5	3	30.334	0.000	-30.334	-0.000	-0.000	0.000
9	10	5	6	3	30.334	0.000	-30.334	-0.000	-0.000	0.000
8	7	6	5	3	30.334	0.000	-30.334	-0.000	-0.000	0.000
9	8	7	6	3	30.334	0.000	-30.334	-0.000	-0.000	0.000
18	12	13	15	3	30.334	0.000	-30.334	-0.000	-0.000	0.000
10	5	6	7	3	30.334	0.000	-30.334	-0.000	-0.000	0.000
16	17	18	12	3	30.334	0.000	-30.334	-0.000	-0.000	0.000
10	9	8	7	3	30.334	0.000	-30.334	-0.000	-0.000	0.000
18	17	16	15	3	30.334	0.000	-30.334	-0.000	-0.000	0.000
16	15	13	12	3	30.334	0.000	-30.334	-0.000	-0.000	0.000
17	16	15	13	3	30.334	0.000	-30.334	-0.000	-0.000	0.000
17	18	12	13	3	30.334	0.000	-30.334	-0.000	-0.000	0.000
9	10	5	4	3	30.334	0.000	-30.334	-0.000	-0.000	0.000
7	6	5	4	3	30.334	0.000	-30.334	-0.000	-0.000	0.000
16	15	13	14	3	30.334	0.000	-30.334	-0.000	-0.000	0.000
18	12	13	14	3	30.334	0.000	-30.334	-0.000	-0.000	0.000

17	18	12	11	3	30.334	0.000	-30.334	-0.000	-0.000	0.000
15	13	12	11	3	30.334	0.000	-30.334	-0.000	-0.000	0.000
10	5	4	2	3	0.000	0.000	0.000	-0.000	-0.000	0.000
6	5	4	2	3	0.000	0.000	0.000	-0.000	-0.000	0.000
13	12	11	10	3	8.786	0.000	-8.786	-0.000	-0.000	0.000
18	12	11	10	3	8.786	0.000	-8.786	-0.000	-0.000	0.000
5	4	2	3	3	2.284	0.000	-2.284	-0.000	-0.000	0.000
5	4	2	1	3	2.284	0.000	-2.284	-0.000	-0.000	0.000
12	11	10	5	3	8.786	0.000	-8.786	-0.000	-0.000	0.000
12	11	10	9	3	8.786	0.000	-8.786	-0.000	-0.000	0.000
19	18	12	13	3	30.334	0.000	-30.334	-0.000	-0.000	0.000
19	18	17	16	3	30.334	0.000	-30.334	-0.000	-0.000	0.000
19	18	12	11	3	30.334	0.000	-30.334	-0.000	-0.000	0.000
14	13	12	11	3	30.334	0.000	-30.334	-0.000	-0.000	0.000
26	11	12	13	3	8.494	0.000	-8.494	-0.000	-0.000	0.000
26	11	10	9	3	8.494	0.000	-8.494	-0.000	-0.000	0.000
26	11	12	18	3	8.494	0.000	-8.494	-0.000	-0.000	0.000
26	11	10	5	3	8.494	0.000	-8.494	-0.000	-0.000	0.000
24	8	7	6	3	30.334	0.000	-30.334	-0.000	-0.000	0.000
24	8	9	10	3	30.334	0.000	-30.334	-0.000	-0.000	0.000
22	6	5	10	3	30.334	0.000	-30.334	-0.000	-0.000	0.000
23	7	6	5	3	30.334	0.000	-30.334	-0.000	-0.000	0.000
27	15	13	12	3	30.334	0.000	-30.334	-0.000	-0.000	0.000
22	6	7	8	3	30.334	0.000	-30.334	-0.000	-0.000	0.000
29	17	16	15	3	30.334	0.000	-30.334	-0.000	-0.000	0.000
28	16	15	13	3	30.334	0.000	-30.334	-0.000	-0.000	0.000
25	9	8	7	3	30.334	0.000	-30.334	-0.000	-0.000	0.000
23	7	8	9	3	30.334	0.000	-30.334	-0.000	-0.000	0.000
29	17	18	12	3	30.334	0.000	-30.334	-0.000	-0.000	0.000

28	16	17	18	3	30.334	0.000	-30.334	-0.000	-0.000	0.000
25	9	10	5	3	30.334	0.000	-30.334	-0.000	-0.000	0.000
27	15	16	17	3	30.334	0.000	-30.334	-0.000	-0.000	0.000
22	6	5	4	3	30.334	0.000	-30.334	-0.000	-0.000	0.000
27	15	13	14	3	30.334	0.000	-30.334	-0.000	-0.000	0.000
29	17	18	19	3	30.334	0.000	-30.334	-0.000	-0.000	0.000
24	8	7	23	3	30.334	0.000	-30.334	-0.000	-0.000	0.000
29	17	16	28	3	30.334	0.000	-30.334	-0.000	-0.000	0.000
28	16	15	27	3	30.334	0.000	-30.334	-0.000	-0.000	0.000
25	9	8	24	3	30.334	0.000	-30.334	-0.000	-0.000	0.000
23	7	6	22	3	30.334	0.000	-30.334	-0.000	-0.000	0.000
25	9	10	11	3	30.334	0.000	-30.334	-0.000	-0.000	0.000
21	4	2	1	3	0.000	0.000	0.000	-0.000	-0.000	0.000
20	4	2	1	3	0.000	0.000	0.000	-0.000	-0.000	0.000
20	4	2	3	3	0.000	0.000	0.000	-0.000	-0.000	0.000
21	4	2	3	3	0.000	0.000	0.000	-0.000	-0.000	0.000
21	4	5	6	3	0.000	0.000	0.000	-0.000	-0.000	0.000
20	4	5	6	3	0.000	0.000	0.000	-0.000	-0.000	0.000
20	4	5	10	3	0.000	0.000	0.000	-0.000	-0.000	0.000
21	4	5	10	3	0.000	0.000	0.000	-0.000	-0.000	0.000
11	10	9	8	3	30.334	0.000	-30.334	-0.000	-0.000	0.000
11	10	5	6	3	30.334	0.000	-30.334	-0.000	-0.000	0.000
11	10	5	4	3	30.334	0.000	-30.334	-0.000	-0.000	0.000

[pairs]

1	5	1
3	5	1
2	6	1
4	7	1

2	10	1
5	8	1
4	9	1
6	9	1
4	11	1
7	10	1
6	11	1
5	12	1
8	11	1
9	12	1
1	20	1
1	21	1
10	13	1
3	20	1
3	21	1
11	14	1
11	15	1
6	20	1
4	22	1
6	21	1
12	16	1
11	17	1
10	18	1
5	23	1
14	16	1
13	17	1
11	19	1
10	20	1
8	22	1

6	24	1
5	25	1
10	21	1
5	26	1
14	18	1
13	19	1
10	22	1
9	23	1
7	25	1
15	18	1
10	24	1
16	19	1
9	26	1
11	25	1
13	26	1
12	27	1
14	27	1
13	28	1
12	29	1
18	26	1
17	27	1
15	29	1
22	23	1
18	28	1
23	24	1
19	29	1
24	25	1
27	28	1
28	29	1

; Include Position restraint file

#ifdef POSRES

#include "posre.itp"

#endif

Section S2. Structure of the diclofenac anion hydrated with two water molecules

6	0.783137	2.118104	-0.065981
6	-0.326020	1.306058	0.263028
7	-0.501816	0.084368	-0.423653
6	-2.662649	-2.837553	0.461681
6	-3.904148	-2.396357	0.011847
6	-4.027976	-1.141284	-0.579912
6	-2.902255	-0.339457	-0.722022
6	-1.629438	-0.727293	-0.258066
6	-1.558167	-2.003040	0.334666
6	-1.227849	1.725758	1.250195
6	-1.047428	2.940983	1.907630
6	0.044464	3.750008	1.596300
6	0.945272	3.326031	0.617597
6	1.774190	1.707291	-1.122748
6	2.803320	0.639595	-0.710428
8	2.728269	0.125556	0.441779
8	3.670194	0.337286	-1.581207
1	0.370330	-0.439019	-0.583892
1	-2.550254	-3.817844	0.925656
1	-4.777770	-3.034876	0.117049
1	-4.991228	-0.789935	-0.950797
17	-3.082902	1.204045	-1.551342
17	-0.007519	-2.573426	0.957482
1	-2.073069	1.093484	1.509220
1	-1.760837	3.247265	2.669823
1	0.194262	4.698993	2.105459
1	1.798976	3.952370	0.364258

1	2.353684	2.594130	-1.466731
1	1.249074	1.302694	-2.022607
8	3.858158	-2.273612	1.178307
1	3.431824	-1.429051	0.898660
1	3.190669	-2.966113	1.001372
8	5.622800	-1.586316	-1.029709
1	4.947164	-0.896487	-1.212657
1	5.244965	-2.063089	-0.269706

Section S3. Structure of the diclofenac anion hydrated with three water molecules

6	0.106570	2.408248	0.083297
6	-0.797132	1.353879	0.346440
7	-0.757438	0.204272	-0.472173
6	-2.230137	-3.179939	0.135755
6	-3.557309	-2.961445	-0.223280
6	-3.964729	-1.703793	-0.662724
6	-3.034345	-0.675158	-0.745436
6	-1.686589	-0.837199	-0.367627
6	-1.326231	-2.125470	0.073007
6	-1.712595	1.468089	1.401503
6	-1.747814	2.615789	2.190716
6	-0.860279	3.662399	1.944441
6	0.055436	3.542830	0.897327
6	1.107877	2.323451	-1.038391
6	2.351311	1.457526	-0.769220
8	2.450233	0.849787	0.333131
8	3.209764	1.414543	-1.699946
1	0.191666	-0.105665	-0.723556
1	-1.896152	-4.159070	0.480247
1	-4.275997	-3.775235	-0.166318
1	-4.997608	-1.522852	-0.961390
17	-3.568991	0.876982	-1.384061
17	0.338228	-2.426243	0.576805
1	-2.397668	0.650190	1.608828
1	-2.467263	2.682986	3.004118
1	-0.879663	4.560957	2.556371
1	0.750623	4.355855	0.694879

1	1.479486	3.341888	-1.295368
1	0.634463	1.915385	-1.964732
8	4.402075	-1.014733	0.814584
1	3.698498	-0.341450	0.652361
1	4.957669	-0.947291	0.014256
8	5.398831	-0.253440	-1.722587
1	4.641409	0.380761	-1.711752
1	6.201652	0.302403	-1.776809
8	3.511452	-3.553084	1.482102
1	2.784631	-3.426744	2.122451
1	3.796247	-2.644401	1.241847

Section S4. Structure of dimer of the diclofenac anion hydrated with four water molecules.

6	3.636398	1.865532	-0.055953
6	2.883020	0.704119	-0.334353
7	3.116537	-0.461363	0.431473
6	1.858024	-3.934274	-0.163390
6	0.563140	-3.835217	0.338472
6	0.108732	-2.629798	0.868160
6	0.960727	-1.533666	0.899773
6	2.269796	-1.573856	0.379750
6	2.679436	-2.812491	-0.148244
6	1.920934	0.723435	-1.351167
6	1.686102	1.881834	-2.086826
6	2.423291	3.035088	-1.825230
6	3.390568	3.010822	-0.818796
6	4.674642	1.878800	1.033762
6	5.991700	1.141003	0.728559
8	6.116839	0.554054	-0.389076
8	6.873522	1.160711	1.629246
1	4.111898	-0.691837	0.557420
1	2.227790	-4.870677	-0.581935
1	-0.094378	-4.700658	0.317382
1	-0.900633	-2.538714	1.268796
17	0.374891	-0.049006	1.638655
17	4.301744	-2.953406	-0.832453
1	1.357823	-0.179752	-1.568283
1	0.923014	1.877951	-2.860316
1	2.243190	3.946300	-2.390249
1	3.967223	3.909027	-0.604455

1	4.268779	1.414968	1.965881
1	4.946344	2.927632	1.291685
6	-4.037764	-0.847674	-1.923858
6	-3.038137	-0.075753	-1.289131
7	-3.428333	1.028045	-0.497696
6	-1.454756	2.847476	2.108391
6	-0.661160	3.671139	1.314631
6	-0.786288	3.636577	-0.071699
6	-1.711040	2.777549	-0.651960
6	-2.507756	1.895081	0.103648
6	-2.346342	1.972033	1.500250
6	-1.688916	-0.411147	-1.458499
6	-1.318479	-1.496273	-2.247905
6	-2.292538	-2.268738	-2.877859
6	-3.637474	-1.935585	-2.704519
6	-5.500425	-0.525640	-1.766366
6	-6.151568	-0.962638	-0.441696
8	-7.388666	-0.724080	-0.324714
8	-5.436660	-1.517564	0.440719
1	-4.275637	0.864391	0.062969
1	-1.366486	2.861596	3.194944
1	0.055280	4.344915	1.777790
1	-0.178371	4.281179	-0.704921
17	-1.905815	2.830040	-2.401321
17	-3.283224	0.890905	2.533863
1	-0.926889	0.172091	-0.954449
1	-0.264110	-1.741896	-2.351708
1	-2.013004	-3.120181	-3.493590
1	-4.406796	-2.531096	-3.193243

1	-6.086620	-1.006552	-2.582536
1	-5.678324	0.573920	-1.855168
8	7.942794	-1.452665	-0.560707
1	7.284300	-0.714176	-0.502779
1	7.407677	-2.264566	-0.672028
8	9.293302	-1.257051	1.880816
1	8.824390	-0.510525	2.302451
1	8.833719	-1.365951	1.021922
8	-6.087646	-1.764887	3.094259
1	-5.837953	-1.651624	2.145794
1	-5.593658	-1.070568	3.574395
8	-8.711478	-1.149606	2.110702
1	-8.270935	-1.015393	1.243292
1	-7.963444	-1.359368	2.698216

Section S5. Structure of dimer of the diclofenac anion hydrated with six water molecules.

6	3.459901	2.064916	0.129144
6	2.774829	0.901085	-0.284209
7	2.965116	-0.296759	0.441281
6	1.784607	-3.723080	-0.503352
6	0.470472	-3.681681	-0.045754
6	-0.026026	-2.526311	0.553160
6	0.807172	-1.425956	0.703560
6	2.140173	-1.411983	0.248158
6	2.588215	-2.595939	-0.365876
6	1.914938	0.954094	-1.388492
6	1.721864	2.144683	-2.084346
6	2.402699	3.297045	-1.696425
6	3.264531	3.241869	-0.599064
6	4.348403	2.048188	1.343988
6	5.692750	1.316562	1.204960
8	6.048580	0.886818	0.072216
8	6.371116	1.198472	2.269207
1	3.973246	-0.536863	0.627321
1	2.183947	-4.617408	-0.982305
1	-0.171534	-4.551289	-0.161325
1	-1.053974	-2.477395	0.911392
17	0.164586	0.001364	1.504696
17	4.232151	-2.658172	-1.008459
1	1.398943	0.051860	-1.704367
1	1.036198	2.166186	-2.927473
1	2.256351	4.232070	-2.231453
1	3.789313	4.141157	-0.281073

1	3.817811	1.574215	2.203963
1	4.590732	3.091799	1.653974
6	-3.967436	-0.821344	-2.225080
6	-3.020887	0.024002	-1.600898
7	-3.484076	1.112584	-0.831011
6	-1.667498	2.936593	1.887516
6	-0.886834	3.816316	1.142913
6	-0.963009	3.811713	-0.247115
6	-1.828742	2.927897	-0.879064
6	-2.615256	1.995421	-0.174469
6	-2.500336	2.039453	1.228789
6	-1.652512	-0.230474	-1.764160
6	-1.213972	-1.297794	-2.542929
6	-2.135361	-2.135232	-3.168580
6	-3.498960	-1.887538	-2.996752
6	-5.445946	-0.599117	-2.048624
6	-6.035206	-1.065960	-0.705155
8	-7.234193	-0.725793	-0.475009
8	-5.320856	-1.759923	0.070534
1	-4.367152	0.916864	-0.292231
1	-1.613802	2.924428	2.976310
1	-0.217131	4.508471	1.647072
1	-0.361773	4.496697	-0.843167
17	-1.956279	3.010832	-2.634020
17	-3.408466	0.887794	2.211210
1	-0.927431	0.399644	-1.261950
1	-0.146437	-1.481164	-2.640115
1	-1.801230	-2.973300	-3.775326
1	-4.229064	-2.537964	-3.475542

1	-6.009403	-1.152162	-2.837353
1	-5.710240	0.477480	-2.166602
8	8.152339	-0.879406	-0.197644
1	7.402127	-0.242063	-0.137328
1	8.461586	-0.936524	0.727765
8	8.508322	-0.490972	2.569756
1	7.728225	0.110134	2.483760
1	8.227237	-1.194222	3.188648
8	7.595517	-3.350344	-1.316447
1	7.750132	-2.477630	-0.892917
1	6.972210	-3.809910	-0.721396
8	-6.086197	-2.296475	2.657974
1	-5.792851	-2.119300	1.732787
1	-6.996503	-1.941522	2.667246
8	-8.509805	-1.046180	1.934841
1	-8.083424	-0.962951	1.046899
1	-9.306349	-1.594915	1.790196
8	-4.475961	-1.300051	4.683302
1	-5.014904	-1.635852	3.934047
1	-3.562337	-1.264000	4.339474
